# Theta burst stimulation of extrastriate body area for body perception in anorexia nervosa: a randomized controlled trial

**DOI:** 10.1038/s41398-026-04235-8

**Published:** 2026-07-01

**Authors:** Rebecca Boehme, Reinoud Kaldewaij, Morgan Frost-Karlsson, Andrew Wold, Adam Enmalm, Isabel Khoure, Jonna Tell, Jessica Käll, Mimmi Salerud, Elin Rimhagen, Charlotte Jackleus, Sara Barsjö, Magnus Thordstein, Per A. Gustafsson, Håkan Olausson, Maria Zetterqvist

**Affiliations:** 1 Center for Social and Affective Neuroscience, Department of Biomedical and Clinical Sciences, Linköping University, Linköping, Sweden; 2 Center for Medical Imaging and Visualization, Linköping University, Linköping, Sweden; 3 Department of Experimental Psychology, Helmholtz Institute, Utrecht University, Heidelberglaan 1, CS Utrecht, The Netherlands; 4 Department of Clinical Neurophysiology in Linköping, Region Östergötland, Linköping, Sweden; 5 Clinical Department of Child and Adolescent Psychiatry in Linköping, Region Östergötland, Linköping, Sweden

**Keywords:** Psychiatric disorders, Neuroscience

## Abstract

Anorexia nervosa is a severe and potentially life-threatening psychiatric disorder characterized by self-starvation, intense fear of weight gain, and a distorted body perception. Treatment remains challenging, and effective interventions for adults are limited. The extrastriate body area (EBA), a cortical region involved in body representation, may contribute to the body perception disturbances. In this double-blind, placebo-controlled, randomized proof-of-concept trial, we investigated the therapeutic potential of targeted theta burst transcranial magnetic stimulation (TMS) of the EBA in patients with anorexia nervosa (*n* = 40). Participants received four weeks of active (*n* = 10) or sham (*n* = 10) TMS combined with body perception training, while a treatment-as-usual group (*n* = 20) and a healthy control group (*n* = 40) served as comparators. Improvements in the primary outcome measure, the Body Shape Questionnaire, differed across groups over time, with active TMS showing faster improvement (after 4 weeks) relative to both control groups, that was sustained at 6 months follow-up. Moreover, active stimulation was associated with changes in EBA responses to self- versus non-self touch, shifting neural activity patterns more closely to those of healthy individuals. These findings provide preliminary evidence that individualized neuromodulation targeting a disorder-relevant neural substrate combined with behavioral training shows potential for effectively recalibrating disturbed body perception. By integrating brain stimulation with behavioral training, this study exemplifies a precision psychiatry approach that links neurobiological mechanisms to personalized therapeutic interventions in anorexia nervosa.

## Introduction

Anorexia nervosa (AN) is a severe, potentially lethal disorder, typically affecting adolescent girls and young women. AN is characterized by self-starvation accompanied by fear of gaining weight and distorted body perception. This body perception disturbance, a central diagnostic criteria for AN [1], seems to be a consequence of patients’ failure to integrate the subjective experience of their own body with actual visual and sensorimotor percepts [2]. It has been suggested that AN patients are stuck in an allocentric perception of their body which is not being updated in spite of contradicting sensory feedback [3]. Targeting body perception disturbances is difficult and altered body perception often persists even in remission [4, 5]. From a precision psychiatry perspective, such enduring distortions highlight the need for individualized interventions grounded in neurobiological mechanisms. Understanding how specific neural systems contribute to maladaptive body representations can inform the development of targeted, mechanism-based treatments rather than one-size-fits-all approaches.

The internal representation or model of one’s body is developed and maintained based on a variety of factors, including sense of agency, sensory side sensations from within the own body (interoception), information about the position of limbs (proprioception), and exteroceptive information (e.g. vision and audition). Differences in motoric [6] and interoceptive tasks [7, 8] are reported in AN. As something in between intero- and exteroception, the sense of touch plays an especially important role in forming the sense of the bodily self [9]. The skin forms the clearest border of the body, and touch is important for learning the difference between self vs. other, starting prenatally. AN patients experience touch from others as less pleasant [10], evaluate vicarious touch as less pleasant [11], and show lower neural involvement of reward structures during social touch [12]. We previously found heightened neural activity to both touch from others as well as self-touch in somatosensory and social processing areas [13]. We interpreted this within the predictive processing framework as a hint that the body is represented or modeled incorrectly (i.e. larger than it actually is). Touch to the body’s actual boundaries could then evoke stronger neural responses due to the conflict between prediction and sensation.

Regarding exteroceptive representation, the visual perception of the own body is studied extensively in AN. Two regions emerge as potentially affected in AN: the extrastriate body area (EBA) and the fusiform body area (FBA) [14]. They are located in lateral-occipital cortex and activate to images of bodies and body parts [15, 16]. The EBA activates more in response to one’s own body images than to those of others [17–19], and plays a role in aesthetic judgement [20, 21]. There is some evidence that EBA functions might be in part lateralized: the right EBA plays a role in self-other-distinction [19, 22–24] while the left EBA might be more involved in social interactions, tools, and hand recognition [25–27]. It has even been suggested that right EBA plays a broader role than static visual body-part representation, contributing instead to sensorimotor integration and self–other distinction [28].

Interestingly, TMS to the EBA in healthy individuals interfered with the aesthetic judgement of bodies (reducing aesthetic sensitivity to body stimuli while increasing liking for bodies of the opposite gender [29, 30]), and selectively biased judgments of one’s own body when evaluated from an allocentric perspective [17]. Specifically, EBA stimulation increased underestimation of one’s own body size when participants judged how they believed others perceived their body, supporting the idea that EBA is involved in the allocentric representation of one’s own body [22, 24]. Furthermore, AN patients display reduced EBA gray matter density [31], altered EBA activation (with the directionality depending on task demands [32–35]) and connectivity [36]. After a three-month body exposure treatment for AN, activation of the EBA in response to own-body images increases compared to pre-treatment [37], indicating that EBA might be involved in the veridical perception of one’s own body.

Identifying such disorder-relevant neural targets provides an opportunity to develop personalized neuromodulatory strategies that directly address the biological mechanisms underpinning psychiatric symptoms. Non-invasive brain stimulation like transcranial magnetic stimulation (TMS) offers such an interventional opportunity. Depending on the frequency, TMS can cause excitation or inhibition in the stimulated brain area. Patterned protocols such as intermittent theta-burst stimulation (iTBS) deliver high-frequency bursts in brief trains and produce lasting changes in cortical excitability with shorter session durations [38]: cortical excitability and plasticity potentials are increased for 30–60 min [39, 40]. Importantly, the excitatory versus inhibitory classification of these paradigms is based largely on motor cortex studies [41] and may not generalize to visual association areas; effects can vary with parameters and individual factors, and reproducibility in non-motor targets is an open question [42, 43]. However, intermittent theta burst TMS has been found to induce plastic changes also in non-motor brain areas [44–46]. This protocol is quicker and employs fewer pulses than classic repetitive TMS, and was selected here to maximize feasibility in a clinical population. We aimed to therapeutically engage the patients during this window of opportunity with a focused body perception training.

Considering previous evidence of reduced EBA activity in AN, we hypothesized that targeting the individually localized right EBA with excitatory theta burst TMS followed by body-perception training could improve body perception disturbances in AN, contributing to better treatment outcomes. We stimulated the right EBA because of its involvement in self-other-distinction and its functional connectivity with right lateralized networks involved in self-related processes [28, 47]. However, there is still no clear consensus about specifically lateralized functions for the EBA.

Our preregistered hypotheses (https://osf.io/z9jv3) included an improvement in our primary outcome measure, the body shape questionnaire [48], for the group receiving a body perception training (BPT) compared to the group receiving treatment as usual (TAU). Within BPT, we expected to find a larger improvement for those receiving active vs. sham TMS. Furthermore, we expected an increase in neural activity in the EBA, which would be more pronounced in the active TMS group. Finally, we expected to find a similar change in our secondary outcome measures (additional questionnaires) and a relationship between EBA neural activity and treatment efficacy.

## Methods

### Participants

**Fig. 1:**
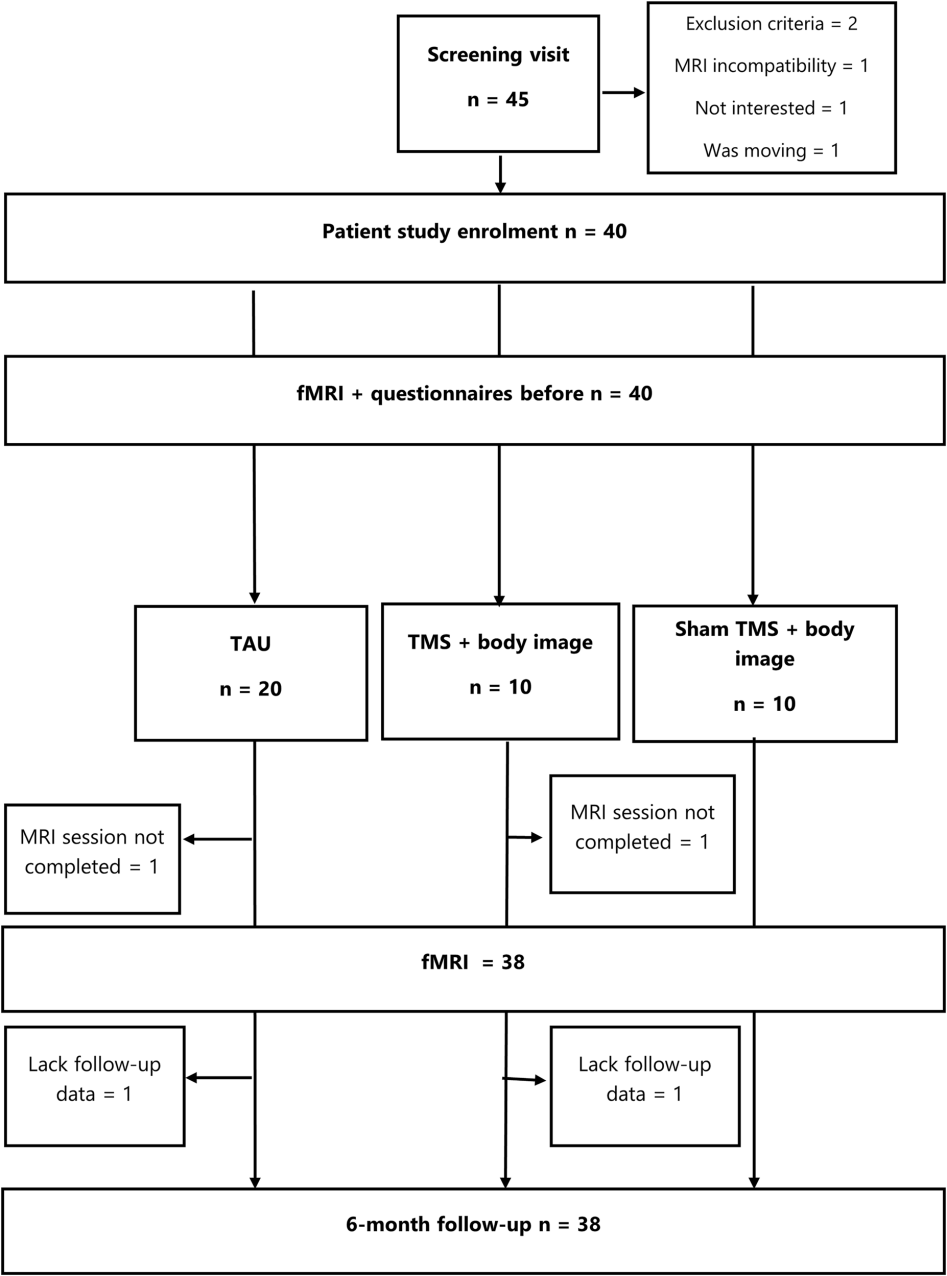
Recruitment and attrition flow chart. (f)MRI (functional) magnetic resonance imaging, TAU treatment as usual, TMS theta burst transcranial magnetic stimulation.

The patient sample (*n* = 40, female) was recruited via the outpatient eating-disorder subunit of the Child and Adolescent Psychiatric clinic and the Psychiatric clinic in Linköping, Region Östergötland. Inclusion criteria were: DSM-5 diagnosis of anorexia nervosa, including atypical and unspecified (restrictive type) with body perception disturbances, 18–35 years of age, BMI ≤ 20 kg/m^2^, and MRI-compatibility (see supplement for more details, Table 1 for patient characteristics, and Fig. 1 for recruitment/attrition). No formal cut-off was used for body perception disturbance. Mean number of weeks between participants’ first visit at the eating disorder units and enrolment in the study was 107.92, SD = 162.39 (*n* = 37).

**Table 1:** Baseline demographic data on clinical AN sample (*n* = 40) presented by treatment allocation: TMS (*n* = 10), sham TMS (*n* = 10), and TAU (*n* = 20).

	TMS *n* (%)	Sham TMS *n* (%)	TAU *n* (%)	Stats (*p-*value)
**Gender**				
Female	10 (100)	10 (100)	20 (100)	
**Age** (*m*, *sd*)	21.10 (3.31)	21.50 (3.87)	21.55 (3.33)	0.83
**Country of origin**				0.02
Born in Sweden	7 (70.0)	10 (100)	20 (100)	
Born in other European country	3 (30.0)	0 (0)	0 (0)	
**Living situation**				0.54
With partner	2 (20.0)	1 (10.0)	6 (30.0)	
Separated	3 (30.0)	1 (10.0)	3 (15.0)	
Single household	0 (0)	1 (10.0)	1 (5.0)	
With parents	3 (30.0)	7 (70.0)	8 (40.0)	
Other	2 (20.0)	0 (0)	2 (10.0)	
**Education**				0.82
Comprehensive school	1 (10.0)	2 (20.0)	4 (20.0)	
Theoretical high-school	7 (70.0)	4 (40.0)	12 (60.0)	
Vocational training	2 (20.0)	3 (30.0)	3 (15.0)	
University	0 (0)	1 (10.0)	1 (5.0)	
**Occupation**				0.30
Working	4 (40.0)	3 (30.0)	2 (10.0)	
Sick-leave	1 (10.0)	0 (0)	2 (10.0)	
Studying	2 (20.0)	4 (40.0)	11 (55.0)	
Unemployed	1 (10.0)	0 (0)	0 (0)	
Not known	2 (20.0)	3 (30.0)	5 (25.0)	
**Body Mass Index** (*m*, *sd*)	18.63 (1.20)	18.58 (1.47)	19.21 (1.27)	0.34
**Medication**				
SSRIs	4 (40.0)	4 (40.0)	8 (40.0)	1.0
Central stimulants	1 (10.0)	0 (0)	1 (5.0)	1.0
Melatonin	2 (20.0)	3 (30.0)	1 (5.0)	0.12
Anxiolytics/Sedative antihistamines	3 (30.0)	2 (20.0)	7 (35.0)	0.90
**NEO-FFI-3** (*m*, *sd*)				
Neuroticism	34.20 (9.57)	31.20 (5.33)	32.60 (6.30)	0.41
Extraversion	21.70 (12.10)	26.10 (7.39)	26.25 (9.49)	0.56
Openness	25.20 (5.33)	26.50 (7.31)	25.85 (8.13)	0.98
Agreeableness	38.50 (5.74)	38.60 (6.29)	36.95 (6.71)	0.78
Conscientiousness	27.40 (9.77)	33.20 (8.36)	30.90 (6.57)	0.25
**AQ** (*m*, *sd*)	23.70 (12.17)	16.40 (6.48)	19.40 (9.68)	0.35
**EQ** (*m*, *sd*)	48.60 (14.03)	53.22 (13.95)	49.60 (14.03)	0.63
**MADRS-S** (*m*, *sd*)	25.20 (10.84)	22.40 (3.87)	19.60 (8.49)	0.19
**SCSS** (*m*, *sd*)	31.90 (9.54)	37.30 (6.62)	32.50 (7.72)	0.17
**EDE-Q** (*m*, *sd*)	12.72 (6.47)	10.10 (8.03)	10.42 (6.72)	0.45
**BSQ** (*m*, *sd*)	143.60 (30.74)	122.90 (32.28)	126.95 (29.82)	0.21
**BAT** (*m*, *sd*)	70.70 (15.15)	63.50 (10.70)	61,75 (17.78)	0.27
**FRS** (*m*, *sd*)				
Think	4.65 (1.11)	4.30 (1.99)	4.05 (1.60)	0.22
Feel	5.30 (1.59)	4.85 (1.68)	4.69 (1.66)	0.50
Ideal	1.75 (0.72)	2.25 (0.86)	2.33 (0.83)	0.15

*TMS* transcranial magnetic stimulation, *TAU* treatment as usual, *AN* anorexia nervosa, *SSRI* selective serotonin reuptake inhibitors, *NEO-FFI-3* neo five-factor inventory personality, *AQ* autism spectrum quotient, *EQ* empathy quotient, *MADRS-S* montgomery åsberg depression rating scale, self-report, *SCCS* self-concept clarity scale, *EDE-Q* eating disorder examination questionnaire, *BSQ* body shape questionnaire, *BAT* body attitude test, *FRS* figure rating scale.

Group differences were analyzed using Fisher’s exact test for categorical data and non-parametric Kruskal-Wallis test for continuous data.

No formal a priori power calculation was conducted, as the study was designed as an exploratory, proof-of-concept trial to assess feasibility and preliminary signals of change rather than definitive clinical efficacy. Sample size was determined by practical and ethical considerations and reflected our nested comparison design, with body perception training (*n* = 20) evaluated against treatment as usual (*n* = 20), and active versus sham TMS examined as subgroups within the training arm.

As a comparison group, healthy control subjects (*n* = 40 female, mean age: 21.9 years) were recruited via flyers, social media, and email advertisement. They were matched to the patients with respect to age. During the first phone contact, volunteers were interviewed to exclude any history of psychiatric illness or substance dependence. BMI for healthy controls was 21.8 (2.4). Mean scores (*n* = 39, due to missing data for one participant) were 19.11 (9.6) on the Body Attitude Test, 0.46 (0.8) for the Figure Rating Scale, and 0.88 (1.5) for the Eating Disorder Examination Questionnaire, indicating low scores for eating disorder symptomatology and body image concerns for controls. Further exclusion criteria were MRI contraindications and any serious health concerns. They participated in a single visit (see below).

### Procedure

**Fig. 2:**
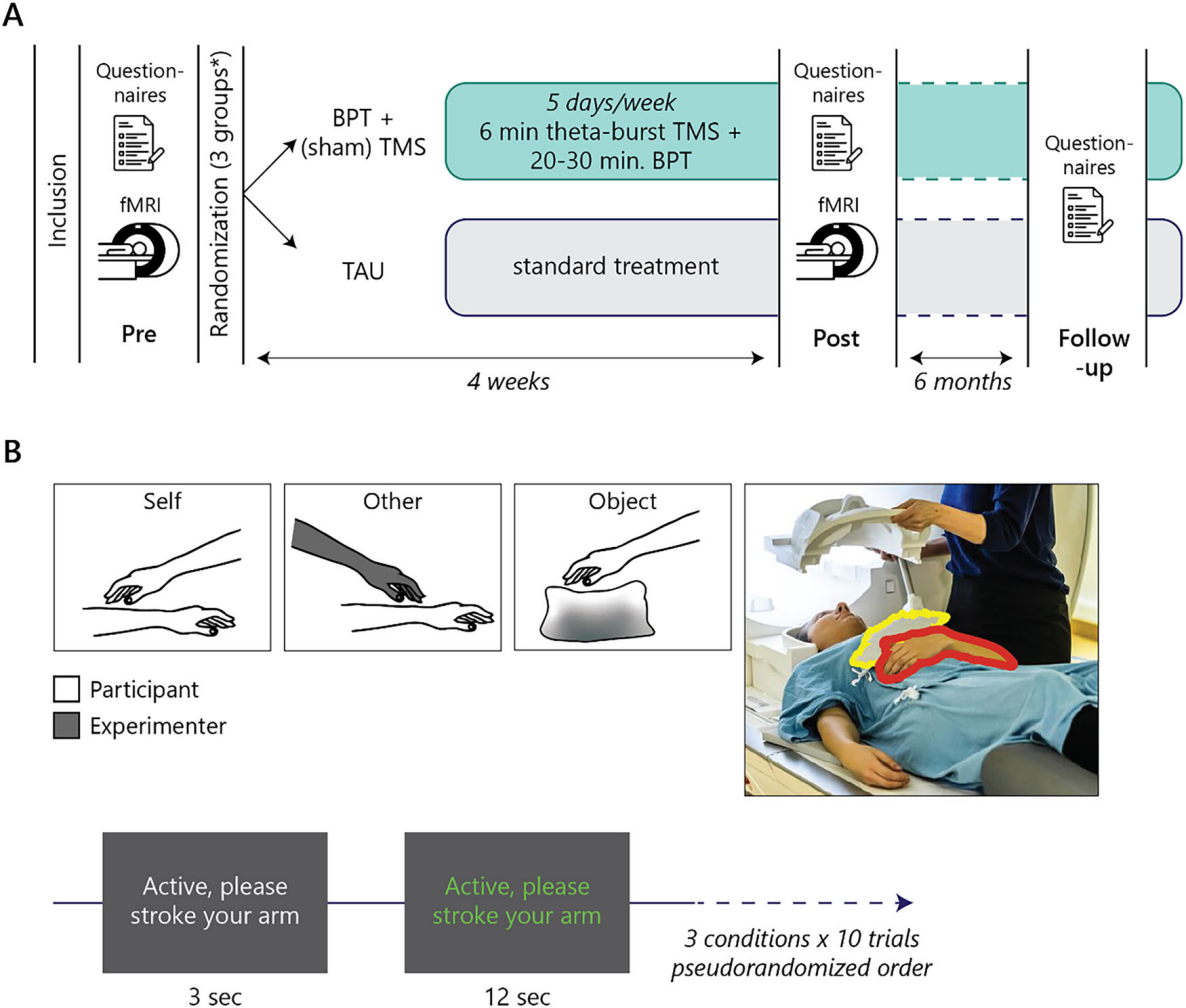
Study procedures. **A** Overview of the study timeline. BPT body perception training, TMS transcranial magnetic stimulation, TAU treatment as usual. * Patients were randomized in three groups: active TMS, sham TMS and TAU. **B** The self-other-touch task [58] consisted of self-touch, other-touch or object-touch (movement control condition) during functional MRI. The position of the arm (red) and cushion (yellow) are outlined on the picture. The tasks consisted of 30 trials, which had a cue phase (white text) and stimulus phase (green text).

This study followed a double-blind, randomized, controlled design. Patients were randomized to treatment as usual (TAU, *n* = 20), body perception training with sham TMS (BPT, *n* = 10) or with active TMS (TMS-BPT, *n* = 10). See Fig. 2A for an overview of the study. Participating staff involved in interaction with the patients and with data analysis were blinded to the TMS-condition, except for the researcher applying the TMS (authors AW, MT) and the study nurses. The unblinded researchers did not interact with the patients outside the TMS-application.

The study protocol was registered at clinicaltrials.gov NCT04213820 “TMS and Body Image Treatment for Anorexia Nervosa” (https://clinicaltrials.gov/study/NCT04213820). Data was collected from September 18^th^ 2019 to April 2nd 2024. Due to covid19-related restrictions during different time-periods (March 2020, December 2020, September to December 2021), seven participants were directly assigned to TAU in order to reduce their time of physical presence in the hospital.

We pre-registered our hypotheses and planned analyses before any formal data analyses (https://osf.io/z9jv3). Blinding was broken after group-statistics had been performed.

In a first experimental visit, all participants answered questionnaires and underwent functional magnetic resonance imaging (fMRI). Participants in the healthy control group followed the same procedure during their single study visit, with fewer questionnaires. Patients in the TAU group continued their treatment at the eating disorder unit and then came back for a second experimental visit with questionnaires and fMRI after four weeks.

Patients in the two intervention groups participated in the BPT over a period of four weeks. They received TMS/sham-TMS for 6 min before participating in the 20–30 min therapist-led intervention (see details below), five days per week, resulting in 20 sessions. Within a week after successfully participating in the treatment (min. 17 out of 20 sessions), patients took part in a second experimental visit with questionnaires and fMRI. Two patients did not want to participate in the second fMRI scan but filled in the questionnaires (TMS-BPT = 1, TAU = 1). Analysis was based on intention to treat.

At a follow-up after 6 months, patients filled in the clinical questionnaires for a third time.

The study and procedures were approved by the Regional Ethical Board of Linköping (2017/443-31, 2018/444-32) and the Swedish Ethical Review Authority (2019-02821), and procedures were performed in accordance to the approved protocol and ethical guidelines and relevant regulations. The participants received oral and written information about the study. Written informed consent was obtained before the initiation of any study procedures.

### Self-report measures

The primary outcome measure to evaluate success of TMS and body perception training was the Body Shape Questionnaire (BSQ; [48]), a 34-item self-report measure of body shape dissatisfaction with established psychometric validity. Total scores range from 34 to 204, with higher scores indicating greater body shape concern; scores <110 are considered within the normal range, scores between 110–140 indicate moderate concern, and scores >140 indicate high body shape dissatisfaction [49]. All treatment groups showed an average score higher than 110 at baseline (Table 1).

In addition, the following clinical measures were collected at experimental visits one and two and at the 6-month follow-up: Body Attitude Test (BAT; [50]), a validated measure of negative body attitudes (range 0–100), with scores >36 indicating clinically significant body dissatisfaction, Eating Disorder Examination Questionnaire (EDE-Q; [51]), Figure Rating Scale (FRS; [52]).

To further characterize the sample, the following measures were collected: Montgomery Åsberg Depression Rating Scale (MADRS-S; [53]), Empathy Quotient (EQ; [54]), Autism spectrum Quotient (AQ; [55]), Neo five-factor inventory personality scale (NEO-FFI-3; [56, 57]), and the Self-Concept Clarity Scale (SCCS) [58].

### Brain imaging

See supplement for details on data acquisition during MRI. The total time in the scanner was maximally 1 h. There were four different tasks: Resting state, a body-image paradigm (to be reported elsewhere), an EBA-localizer task, and a self-other-touch task [59].

#### Resting state (12 min)

Participants were asked to relax and visually fixate on a cross presented through scanner-compatible goggles.

#### EBA-localizer task (8 min)

Patients were presented with 8 blocks of body images and 8 blocks of landscape images (10 per block, each 2 s, pseudorandomized order). Between blocks, a fixation cross was displayed for 10 s.

#### Self-other-touch task (13 min)

The previously established self-other-touch task [59] (see Fig. 2B) contained three conditions (3 s cue, 12 s touch, 10 repetitions each, in random order): self-touch (touching the own arm), other-touch (touch on the arm by the experimenter), and object-touch (touching a pillow by the participant, movement control condition). All touch occurred on the left forearm. Participants were instructed to use slow, soft stroking typically perceived as being pleasant and effective in evoking C-tactile fiber activity [60].

### TMS

TMS was performed on the individually localized right EBA with a figure-eight coil driven by a Super Rapid Transcranial Magnetic Stimulator (MagVenture MagPro X100 incl. MagOption, MagVenture, Farum, Denkmark). During the first session, the patients´ structural MRI was co-registered to their head position, which was tracked in real time using a Nexstim ITMS Navigation system (Nexstim Ltd., Helsinki, Finland) allowing us to navigate coil position relative to the target (i.e. the EBA). The location was marked on a neoprene cap, which was then used to target the location during the following sessions of this participant. Right EBA was identified based on the individual patients’ fMRI data. For this purpose, statistical fMRI analysis (see below) was performed on the non-normalized images. Maps were thresholded at *p* < 0.05 or higher if the peak activation was clearly visible. The activation map was overlayed over the individual T1 and the local maximum within the approximate location of the EBA in right lateral occipital lobe was identified.

The subject’s resting-motor threshold was determined by the minimum intensity of pulses over the motor cortex that a visible thumb twitch in at least 5 out of 10 consecutive trials. This approach has been widely used in clinical and exploratory TMS studies [61] and was considered sufficient for the purpose of safely individualizing stimulation intensity. The same procedure was applied consistently across all participants and groups. Electromyographic recordings were not obtained. To maintain blinding, participants were informed that TMS sensations can differ across scalp locations and that motor threshold determination may feel stronger than the subsequent experimental stimulation, which was delivered below motor threshold; however they were not asked to guess stimulation conditions. Then, intermittent theta burst stimulation was administered consisting of bursts containing 3 pulses at 50 Hz and an intensity of 90% of the resting-motor threshold repeated at 200 ms intervals (i.e., at 5 Hz) and 10 s between burst interval (i.e. 2 s stimulation followed by 8 s rest) for a total of 190 s (600 pulses).

In the sham condition, the stimulator was tilted transversely so that the electromagnetic pulses did not stimulate the brain. The participants had no previous TMS experience and therefore no reference for the typical sensations that TMS produces.

### Body perception training

During the 30 min following directly after the TMS treatment, the patients participated in one of five different body perception training sessions focusing on body perception (psychoeducation, drawing of estimated and actual body size, estimating the size of different body parts using a piece of string, or different sized hula hoop rings, and adjusting the size of a computerized morph of their body; see supplement for detailed description). The five sessions were repeated in random order during each week. Together with a therapist, the patients explored and estimated their body size and discussed the accuracy when confronted with their actual size. The therapist also discussed cognitive, emotional, and perceptual aspects of the exercises with the patient.

### Analysis

#### Task fMRI

Data was analyzed using statistical parametric mapping (SPM12; Wellcome Department of Imaging Neuroscience) in Matlab R2015b (MathWorks). See supplement for pre-processing details.

For statistical analyses of the blood oxygen level dependent (BOLD) response, the general linear model approach was used. We used the FAST-option [62] due to the short repetition time, which improves autocorrelation modeling performance [63]. Realignment parameters were added as regressors-of-no-interest. The first temporal derivative of motion parameters in x,y,z-directions and a regressor censoring volumes with more than 1 mm volume-to-volume movement [64] were added in addition to improve movement correction.

For the EBA task, the regressors of interest were the body- and landscape-blocks. The body condition was contrasted with the landscape condition at the subject level. For the touch task, the regressors of interest were the self-, other-, and object-touch conditions, and regressors of no interest were: the cue phase (3 s) and the motion after the active conditions (1 s after self-touch and object touch conditions when the participants moved their hand back to the resting position). Self-touch was contrasted with object-touch to account for arm movement effects. Subsequently, other-touch was contrasted with the movement-corrected self-touch contrast at the subject level.

For both tasks, the main contrasts of interest (body vs. landscape for the EBA, other- vs. self-touch for the touch-task) were entered into three different group-level models. First, a two-sample t-test comparing patients (all treatment groups) and controls, pre-treatment. Second, a flexible factorial ANOVA with main effects of subject, group (BPT vs. TAU), and time (pre- and post-treatment), as well as the interaction between group and time. Third, within the BPT group, a flexible factorial ANOVA with main effects of subject, group (TMS vs. sham), and time, as well as the interaction between group and time.

At the whole brain-level, statistical significance was assessed at a family-wise-error (FWE) corrected level of p < 0.05 (peak-level). Small-volume-corrected analyses were performed for the bilateral EBA and right FBA. The right FBA was added as exploratory region of interest that was not pre-registered, after we observed that the activated clusters extended into this region, and given that the right FBA is strongly implicated in body perception [65, 66]. Masks for these regions were created by drawing 18 mm spheres around the peak voxels from the meta-analytic NeuroSynth tool (neurosynth.org), keyword ‘body’ (see supplement, Fig. S1 for a visualization of these masks). These spheres were masked with a standard brain mask (FMRIB Software Library [67]) to exclude out-of-brain voxels. For the touch-task, small-volume-corrected effects were also assessed in the right anterior cingulate cortex, right superior temporal gyrus, and right insula, in line with previous work [59, 68].

For each participant, additional individual masks were created within the bilateral EBA and right FBA masks. An 8-mm sphere was drawn around the peak voxel coordinates for the body vs. landscape contrast at the individual level. These masks were used as seed regions in the resting state fMRI analyses described below.

#### Baseline demographics

Between-group differences in sample characteristics were analyzed using crosstabs, Fisher’s exact test for categorical data and both one-way ANOVA and non-parametric test Kruskal-Wallis for continuous data.

#### Questionnaires

Questionnaires were analyzed using JASP (JASP Team (2024) Version 0.19.1). For each questionnaire, three repeated measure ANOVAs were run, with time as within-subject factor in each case and the following grouping factors: (1) BPT (both TMS groups combined) vs. TAU, (2) TMS-BPT vs. sham-BPT, and (3) all three groups (TMS-BPT vs. sham-BPT vs. TAU). Significant main and interaction effects were followed up by post-hoc comparison corrected by the Holm method.

## Results

### Effect of body perception training and TMS

#### Body shape questionnaire (primary outcome)

Mean scores for all measures, groups, and timepoints are shown in Table 2. BSQ scores improved significantly over time (main effect of Time *F*(2,72) = 11.42, *p* < 0.001, *η²*_*p*_ = 0.241). The general effect of the body perception training (i.e. combining sham and active TMS groups) compared to TAU was non-significant (pre-post-6month: Time*Group *F*(2,72) = 2.90, *p* = 0.062, *η²*_*p*_ = 0.074).

**Table 2:** Mean scores across groups and timepoints for primary (BSQ) and secondary outcomes.

	TMS	Sham TMS	TAU
	*m (sd)*	*m (sd)*	*m (sd)*
**BSQ**			
Pre	143.60 (30.74)	122.90 (32.28)	126.95 (29.82)
Post	112.00 (37.46)	108.60 (34.54)	119.80 (31.27)
6-months	102.67 (27.63)	121.30 (41.98)	105.11 (32.93)
**BAT**			
Pre	70.70 (15.15)	63.50 (10.70)	61.75 (17.78)
Post	52.40 (19.60)	54.60 (15.45)	61.45 (18.18)
6-months	51.78 (16.58)	58.60 (19.71)	51.68 (20.11)
**EDE-Q**			
Pre	12.72 (6.47)	10.10 (8.03)	10.42 (6.72)
Post	10.72 (6.62)	7.13 (3.91)	8.33 (4.80)
6-months	6.62 (4.01)	6.27 (4.01)	6.35 (4.40)
**FRS-Think**			
Pre	4.65 (1.11)	4.30 (1.99)	4.05 (1.60)
1-week	4.35 (1.36)	4.15 (2.16)	4.14 (1.62)
2-weeks	4.15 (1.56)	4.10 (1.88)	4.23 (1.63)
3-weeks	4.20 (1.42)	4.10 (2.09)	4.18 (1.73)
Post	3.75 (1.36)	4.00 (1.98)	4.08 (1.71)
6-months	3.78 (0.80)	3.95 (2.05)	3.92 (1.54)
**FRS-Feel**			
Pre	5.30 (1.59)	4.85 (1.68)	4.69 (1.66)
1-week	4.80 (1.67)	4.80 (1.95)	4.51 (1.71)
2-weeks	4.63 (1.66)	4.48 (1.74)	4.44 (1.76)
3-weeks	4.73 (1.85)	4.80 (1.87)	4.47 (1.78)
Post	4.45 (1.71)	4.43 (1.78)	4.35 (1.88)
6-months	4.22 (1.23)	4.65 (1.86)	4.39 (1.60)
**FRS-Ideal**			
Pre	1.75 (0.72)	2.25 (0.86)	2.33 (0.83)
1-week	2.00 (0.71)	2.15 (0.94)	2.44 (0.66)
2-weeks	2.00 (0.71)	2.40 (0.91)	2.50 (0.71)
3-weeks	2.20 (0.68)	2.45 (1.04)	2.58 (0.79)
Post	2.20 (0.82)	2.45 (1.04)	2.72 (0.62)
6-months	2.44 (0.98)	2.55 (0.80)	2.77 (0.83)
**BMI**			
Pre	18.63 (1.20)	18.58 (1.47)	19.21 (1.27)
Post	18.71 (1.50)	18.78 (1.83)	19.33 (1.46)
6-months	19.27 (2.29)	18.98 (1.88)	19.61 (1.28)

*BSQ* body shape questionnaire, *BAT* body attitude test, *EDE-Q* eating disorder examination questionnaire, *FRS* figure rating scale, *BMI* body mass index.

**Fig. 3:**
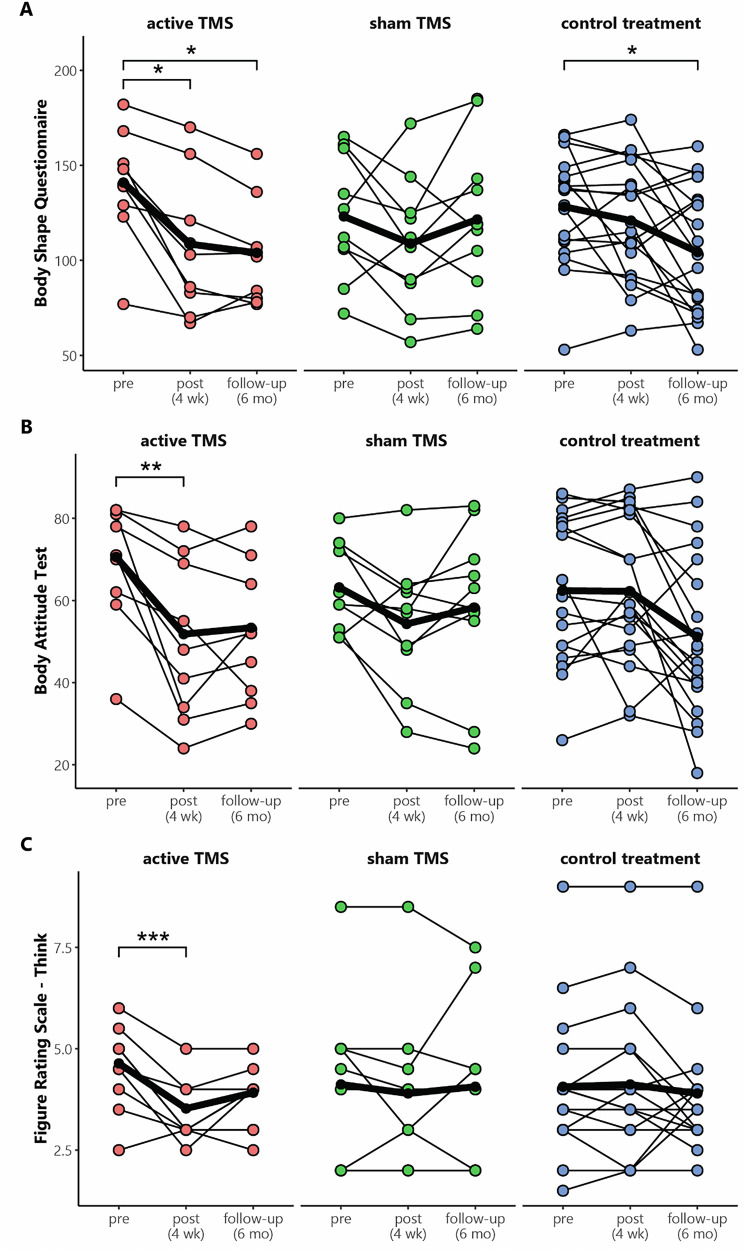
Questionnaire scores across treatment groups and timepoints. **A** Body Shape Questionnaire. **B** Body Attitude Test. **C** Figure Rating Scale – Think. Colored dots represent individual values; thick black lines represent mean values. * *p* < 0.05, ** *p* < 0.01, *** *p* < 0.001.

When comparing all three groups (TMS-BPT, sham-BPT, TAU), improvements differed between groups and time points (interaction Time*Group *F*(4,70) = 3.62, *p* = 0.010, *η²*_*p*_ = 0.17, Fig. 3A). Post-hoc tests suggested that this was driven by a faster and longer lasting improvement in the TMS-BPT group (Pre vs. post: *M*_*diff*_ = 32.44, *t* = 3.83,*p*_*holm*_ = 0.018, 95% CI [3.00, 61.89], Cohen’s *d* = 0.99, 95% CI [0.002, 1.99]; Pre vs. follow-up: *M*_*diff*_ = 37.00, *t* = 3.8, *p*_*holm*_ = 0.019, 95% CI [3.21, 70.79], Cohen’s *d* = 1.13, 95% CI [−0.004, 2.27]), while there were no significant differences between time points in the sham-BPT group (Table S1) and only a significant improvement for Pre vs. follow-up in the TAU group (*M*_*diff*_ = 23.84, *t* = 3.56, *p*_*holm*_ = 0.037, 95% CI [0.58, 47.10], Cohen’s *d* = 0.73, 95% CI [−0.044, 1.505]). All other pairwise comparisons rendered a p-value > 0.05 (Table S1).

This was further supported when comparing the two BPT groups directly: The active TMS group showed a longer-lasting decrease in BSQ scores than the sham-group (interaction Time*TMS-Group *F*(2,34) = 4.80, *p* = 0.015, *η²*_*p*_ = 0.22).

#### Other clinical measures

Comparing the BPT (combined TMS- and sham) and TAU groups revealed a significantly larger improvement for BPT regarding BAT scores and FRS cognitive sub-scores after 4 weeks (pre-post: BAT: *F*(1,38) = 13.57, *p* < 0.001, *η²*_*p*_ = 0.263; FRS: *F*(1,37) = 5.06, *p* = 0.031, *η²*_*p*_ = 0.12, Fig. 3B, C), and an interaction effect (Time*Group, BAT: *F*(1,38) = 12.43, *p* = 0.001, *η²*_*p*_ = 0.246; FRS-think *F*(1,37) = 6.01, *p* = 0.019, *η²*_*p*_ = 0.14, Fig. 3B, C), indicating a stronger decrease for the BPT group.

When comparing all three groups (TMS-BPT, sham-BPT, TAU, Fig. 2B), BAT decreased over time (*F*(2,70) = 12.47, *p* < 0.001, *η²*_*p*_ = 0.26) and showed an interaction effect (Time*Group *F*(4,70) = 4.12, *p* = 0.005, *η²*_*p*_ = 0.19). Holm-corrected post-hoc tests revealed that this was driven by Pre-Post differences in the TMS-BPT group (*M*_*diff*_ = 18.78, *t* = 4.75, *p*_*holm*_ = 0.001, 95% CI [5.04, 32.52], Cohen’s *d* = 1.08, 95% CI [0.171, 1.981]; see Table S2 for all post-hoc comparisons). FRS and EDE-Q showed no main or interaction effects of time and group (see supplement).

There were no adverse events related to TMS or BPT.

### Task-based fMRI

The localizer task activated the EBA, however, the peak was found in the visual cortex (anorexia patients: MNI(x,y,z) = 26, −90, 20; healthy controls MNI(x,y,z) = 10, −92, 8) and activity extended into the FBA (Fig. S1). There was no difference in activation between patients and controls. Analysis of right EBA activation within individualized masks (see methods) revealed no group*time interaction for the activity within this region (*F*(2,70) = 0.209, *p* = 0.813, *η²*_*p*_ = 0.012). There was also no significant overall relationship between right EBA activity and BSQ at baseline (*r*(38) = -0.112, *p* = 0.504), or between right EBA activity change and BSQ change over time (*r*(38) = 0.146, *p* = 0.382).

**Fig. 4:**
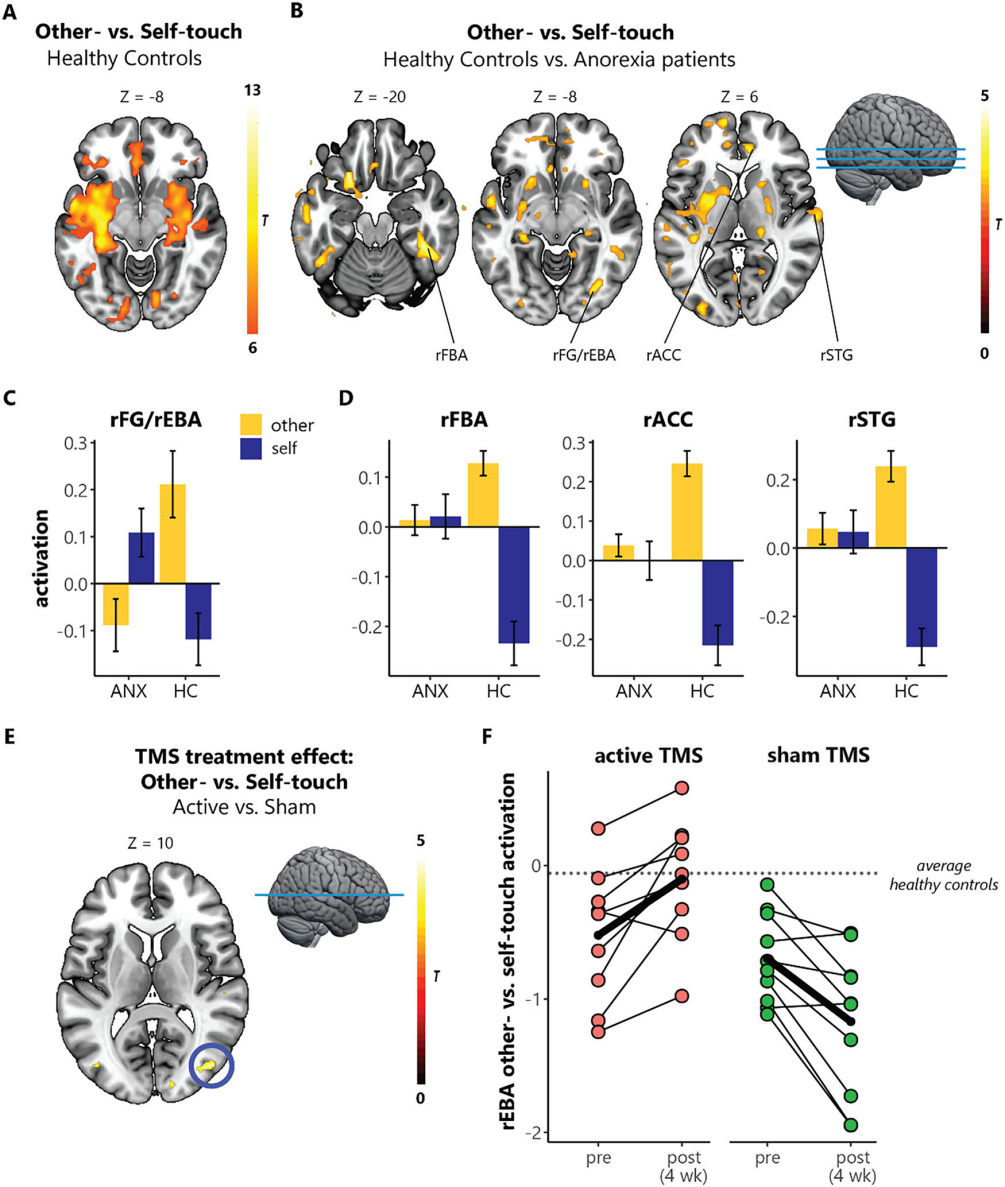
Self-other touch task. **A**. Healthy controls showed increased activation for other- compared to self-touch in regions including the insula, superior temporal gyrus (STG), and anterior cingulate cortex (ACC). Contrast image (other-touch > self-touch) thresholded at family-wise-error corrected *p*-value < 0.05 at whole-brain level. **B** Compared to healthy controls, pre-treatment anorexia patients showed relatively less activation for other- vs. self-touch in the right fusiform gyrus (rFG)/right Extrastriate Body Area (rEBA), right Fusiform Body Area (rFBA), rACC, and rSTG. Small-volume-corrected analyses, contrast image ([HC: other-touch > self-touch] > [ANX: other-touch > self-touch]) thresholded at *p* < 0.001 uncorrected. **C** Compared to healthy controls, *p*re-treatment rFG/rEBA activation (mean beta-values from cluster depicted in panel B) was inversed in anorexia patients, with relatively more activation in response to self-touch and less activation in response to other-touch. **D** Compared to healthy controls, pre-treatment rFBA, rACC, and rSTG other-self differentiation was diminished in anorexia patients (mean beta-values from cluster depicted in panel B) **E** Transcranial magnetic theta burst stimulation (TMS) increased activation in the rEBA for other- vs. self-touch. Small-volume-corrected analysis, contrast image ([POST: [active TMS: other-touch > self-touch] > [passive TMS: other-touch > self-touch]] > [PRE: [active TMS: other-touch > self-touch] > [passive TMS: other-touch > self-touch]]) thresholded at *p* < 0.001 uncorrected **F** For all but one participant receiving active TMS, rEBA activation for other- vs. self-touch increased from pre- to post-treatment, in descriptive terms on average approaching the activation level of healthy controls. See supplemental Fig. S2 for descriptive plots per treatment group and touch condition. T = T-value, Z = MNI coordinate. Image in neurological orientation.

For the self-other-touch task, AN patients showed stronger activation for self-touch compared to other-touch in the right fusiform gyrus (FG)/EBA at baseline. This pattern was opposite in the healthy controls (see Fig. 4B, C). Compared to controls, patients showed a smaller difference in activation between other-touch and self-touch in the right STG and right ACC, two regions important for self-other differentiation of touch in healthy participants [59], as well as in the right FBA (see Fig. 4B, D). See Table S3 for an overview of the significant activations within these clusters.

At the post-treatment timepoint, the two BPT subgroups showed changes of EBA activity in opposite directions (exploratory analysis; Time*Group interaction MNI_xyz_ = 38, −80, 10, *T* = 4.84, *p*(SVC) = 0.045; Fig. 4E note that this a different cluster than the one showing a difference at baseline; see Fig. S3 for illustration). The difference in EBA activity between self-touch and other-touch became smaller in the BPT-TMS group, while the BPT-sham group displayed the opposite: an increase in difference between the two conditions (Fig. 4F). This effect was especially driven by the self-touch condition: activation in the EBA during self-touch decreased in the TMS-BPT group (thereby becoming more similar to controls) while it increased in the sham-BPT group (Fig. S2).

## Discussion

TMS of the EBA in combination with body perception training over the course of four weeks improved body perception concerns in this sample of AN patients. The effect was accompanied by specific changes in EBA activation in a touch paradigm evaluating self-other-distinction. Additional measures of symptom improvement showed a comparable directionality. Given the small sample size and exploratory nature of the study, all findings should be interpreted as preliminary and hypothesis-generating.

The Body Shape Questionnaire (BSQ) assesses clinically meaningful body dissatisfaction, with established severity thresholds in anorexia nervosa. Although improvements over time were also observed in the treatment-as-usual group, indicating that the typical treatment the patients receive at the eating disorder unit is effective, these changes were slower than those seen in the active TMS + BPT group. The active TMS group showed initial symptom reductions after 4 weeks of treatment and they persisted at six-month follow-up, when they were on par with the treatment as usual group, suggesting a durable effect. Previous studies using TMS for depression also find sustained effects for 3–12 months [69–71]. One possible interpretation is that the lasting improvement observed in the active TMS group may relate to TMS-associated changes in EBA activity, although causal inferences cannot be drawn from the present data. While the study was not powered to establish minimal clinically important differences, the convergence of BSQ, BAT, and related measures in the active TMS group supports the presence of a clinically relevant signal that warrants further investigation in larger trials.

The inclusion of both a sham stimulation condition and a treatment-as-usual group allows partial dissociation of intervention components. While the TAU group captured symptom change over time in the absence of structured body perception training, the sham-TMS + BPT condition provided an estimate of behavioral and nonspecific treatment effects. Within this framework, the faster and more sustained reduction in BSQ scores observed in the active TMS + BPT group suggests an incremental benefit of neuromodulation beyond behavioral training alone. However, the present design does not permit conclusions regarding the independent efficacy of TMS or BPT in isolation, nor can it determine whether TMS primarily facilitates behavioral relearning or would be sufficient to induce comparable symptom change without concurrent training. Future factorial designs will be required to disentangle additive versus interactive effects.

Contrary to our hypothesis, there was no difference in activation in the EBA localizer task between controls and patients. Our task evoked peak activation in the FBA, not the EBA, potentially because it employed pictures of full bodies, not body parts [72] suggesting that it was not sensitive enough to evoke the expected response. In addition, it only showed the bodies of *other* people, which might not engage processes specifically related to *own* body perception. EBA connectivity during rest, however, was different for AN patients and controls.

We found differential effects between treatment groups in the EBA during the self-other-touch task. The EBA integrates multimodal signals and is involved in processing tactile body-related sensations, e.g. in the embodiment of a rubber hand [73, 74] and in the enhancement of touch when viewing it [75]. Already in infants, seeing touch activates the EBA [76]. Our participants did not see the touch, as they were viewing a screen through googles. However, visual input might not be necessary, since EBA activates when haptically exploring body parts [77] and in blind people, suggesting a supra-modal role not fully dependent on visual input [78].

Both self- and other-touch involve processes relating to representations of the own body [9, 79]. Here, the active TMS group showed a shift in EBA activation during self-touch relative to other-touch toward the pattern observed in the control group. Consistent with previous work, controls exhibited no or minimal EBA activation during self-touch [59], whereas AN patients showed increased EBA activation during self-touch and deactivation during other-touch at baseline. On possible interpretation of this pattern is that elevated EBA responses during self-touch in AN could reflect atypical processing of self-related bodily signals. Self-generated sensations are typically attenuated when they match prior expectations [80–82]. Heightened neural responses might indicate that sensory input deviates, causing prediction errors, or that attenuation of self-produced sensations fails due to other underlying causes. Although the EBA is not established as a primary locus of tactile processing, it plays a well-documented role in the integration of visual and body-related information. Altered EBA engagement during self-touch might therefore by consistent with reduced or inefficient multisensory integration. This could affect updating of representations of the own body based on tactile signals, and could explain aversiveness of touch in AN observed previously [83]. However, this interpretation remains speculative, as the present study did not include computational or behavioral measures directly indexing prediction or attenuation.

Following active TMS, the reduction in differential EBA responses to self- versus other-touch suggests a shift toward a more typical pattern of body-related processing. In contrast, participants receiving sham stimulation showed increased EBA responses during self-touch over time, possibly reflecting different engagement strategies or nonspecific training effects that were not associated with sustained clinical improvement, as this group’s values did not differ significantly from baseline after four weeks, only showed a descriptive small dip, and returned to baseline levels after 6 months. Alternative explanations, including changes in attentional allocation, salience of self-related stimuli, or task strategy, remain plausible. Future studies incorporating computational modeling or explicit measures of sensory prediction and attenuation will be necessary to test underlying mechanisms directly.

This study is one of the few that have used TMS as a treatment or treatment add-on for AN. Most prior neuromodulation studies have targeted prefrontal or insular regions and primarily reported effects on BMI, eating-related cognitions, or affective symptoms [84–87]. Meta-analytic evidence suggests modest efficacy of these approaches [84], although effects on body image specifically are inconsistent. By contrast, the present study targeted a visual body-representation region and focused on perceptual outcomes rather than weight or eating behavior. While direct comparisons are limited by differences in targets and outcome measures, the sustained improvement in body perception observed here suggests that perceptual mechanisms may represent a complementary therapeutic entry point rather than an alternative to existing approaches.

We were trying to employ a potential window of opportunity where the hypothesized increased plasticity might contribute to improved learning or higher updating potential [88, 89]. Since we found more robust improvements in the TMS group compared to the sham group, there is preliminary support for this idea. It might be important which activities and thoughts a patient engages in during this sensible time window.

### Limitations

The relatively small sample sizes of the active and sham TMS groups (*n* = 10 each) limit the generalizability and statistical power of the present findings. Because of the small group sizes, effect size estimates are likely to be unreliable. Future studies with larger and more diverse samples are needed to confirm the robustness of these effects and to assess individual variability in treatment response – a key consideration for precision psychiatry approaches. While we pre-registered an expected effect on EBA activity, this was specified for the EBA localizer task rather than the touch-task used here. Thus, the current neural findings only partially align with our preregistered hypotheses but nevertheless support the role of the EBA in body perception processing.

Some limitations to the chosen TMS methodology need to be considered: Motor threshold was determined using visual observation of thumb movements rather than EMG recordings, which may have reduced the precision of stimulation intensity estimation, although this procedure was applied consistently across all participants. We stimulated only the right EBA and only used one stimulation protocol (intermittent theta burst stimulation), so our results cannot be generalized to left EBA or other stimulation parameters. We do not claim superiority of the chosen location of protocol over other set-ups. While intermittent theta burst stimulation reliably modulates excitability in motor and some prefrontal regions, evidence for robust effects in visual association cortex is limited. Studies of theta burst TMS applied to primary visual cortex have reported minimal changes in measures of cortical excitability and connectivity following stimulation [90], and systematic reviews highlight that effects outside traditional motor and prefrontal targets are heterogeneous and dependent on baseline neural state and network connectivity [91]. Thus, assumptions of lasting excitatory effects in higher-order visual areas must be made cautiously, and our findings in EBA should be viewed within this context of empirical uncertainty.

## Conclusion

This study provides preliminary proof-of-concept evidence that targeted theta burst stimulation of the extrastriate body area, combined with focused body perception training, is feasible and may beneficially modulate body perception in patients with anorexia nervosa. The differential neural and behavioral responses observed between active and sham TMS groups suggest that individualized neuromodulation may engage distinct mechanisms contributing to sustained therapeutic effects. These findings advance the development of biologically informed, personalized interventions in psychiatry by demonstrating how specific neural targets can be leveraged to recalibrate maladaptive perceptual processes. Future work integrating neuroimaging, computational modeling, and patient-specific neural profiling could further refine such precision-based treatment strategies for anorexia nervosa and related disorders.

## Supplementary information

Supplement

## Data Availability

Data cannot be shared publicly since this was not included in the informed consent and the ethics application.
