# Supplementary material for: Theta burst stimulation of extrastriate body area for body perception in anorexia nervosa: a randomized controlled trial
Source: Transl Psychiatry. 2026 Jul 1;16:324. doi: 10.1038/s41398-026-04235-8 (PMC13328705; doi:10.1038/s41398-026-04235-8)
Supplement: Supplementary file 1 — Supplement [file 41398_2026_4235_MOESM1_ESM.docx]

**Supplement to:**

**Theta burst stimulation of extrastriate body area for body image perception in anorexia nervosa: A Randomized Controlled Trial**

Rebecca Boehme*^1,2^, Reinoud Kaldewaij*^1,2,3^, Morgan Frost-Karlsson^1^, Andrew Wold^4^, Adam Enmalm^1,2^, Isabel Khoure^5^, Jonna Tell^5^, Jessica Käll^5^, Mimmi Salerud^5^, Elin Rimhagen^5^, Charlotte Jackleus^5^, Sara Barsjö^5^, Magnus Thordstein^4^, Per A Gustafsson^1,5^, Håkan Olausson^1,4^, Maria Zetterqvist^1,5^

^1^ Center for Social and Affective Neuroscience, Department of Biomedical and Clinical Sciences, Linköping University, 58185 Linköping, Sweden

^2^ Center for Medical Imaging and Visualization, Linköping University, 58185 Linköping, Sweden

^3^ Department of Experimental Psychology, Helmholtz Institute, Utrecht University, Heidelberglaan 1, 3584, CS, Utrecht, The Netherlands

^4^ Department of Clinical Neurophysiology, Linköping University Hospital, 58185 Linköping, Sweden

^5^ Clinical Department of Child and Adolescent Psychiatry in Linköping, Region Östergötland, 58185 Linköping, Sweden

# Overview Content

[Overview Content](#_Toc209614096)

[Supplementary Methods](#_Toc209614097)

[Participants](#_Toc209614098)

[MRI data acquisition](#_Toc209614099)

[MRI data preprocessing](#_Toc209614100)

[Extrastriate body area and fusiform body area mask definition](#_Toc209614101)

[Resting state analysis](#_Toc209614102)

[Supplementary Results](#_Toc209614103)

[Effect of body perception training and TMS on clinical outcome](#_Toc209614104)

[*Body shape Questionnaire (primary outcome)*](#_Toc209614105)

[*Body Attitude Test*](#_Toc209614106)

[*EDE-Q*](#_Toc209614107)

[*Figure rating scale*](#_Toc209614108)

[*BMI*](#_Toc209614109)

[EBA localizer task](#_Toc209614110)

[Self-other-touch task](#_Toc209614111)

[Resting state](#_Toc209614112)

#

# Supplementary Methods

## Participants

Patients were informed about the study by clinical staff, and those interested were contacted by a research nurse not involved in the patient’s treatment. Patients had to be free of psychotropic medications or on stable (at least three months on the same dose) medication with antidepressants (SSRIs); on-demand use of anxiolytics, hypnotics, and treatment with central stimulants (if possible, paused on MRI investigation day and during iTMS treatment) were accepted. Further, inclusion depended on the judgement of the clinical physician if participation was in the best interest of the patient. Exclusion criteria: schizophrenia or psychotic disorder, bipolar disorder, and/or alcohol/drug dependence, ongoing treatment with antipsychotics or tricyclic antidepressants, previous severe head injury, seizures, birth before 33 weeks of gestation, hearing impairment, earlier epilepsy or seizure of other causes (other than fever cramps in childhood), claustrophobia, pregnancy, and cognitive disability.

## MRI data acquisition

Before entering the MRI scanner, participants familiarized themselves with the tasks in a mock scanner (PST MR Simulator System, BlindSight GmbH, Germany), where they also trained to minimize head movements (MoTrak Head Motion Tracking System, Psychology Software Tools, PA, USA).

Functional neuroimaging data were acquired in a 3T scanner (Prisma, Siemens, Germany). A standard T1 structural scan for subsequent co-registration to functional scans was obtained (repetition time = 2300 ms; echo time = 2.36 ms; flip angle = 8º; field of view = 288 x 288 mm2; voxel resolution = 0.87 x 0.87 x 0.90 mm3), followed by functional scans during the tasks (repetition time: 1030 ms; echo time: 30 ms; slice thickness: 3 mm; matrix size: 64 × 64; field of view: 192 × 192 mm2; in-plane voxel resolution: 3 mm2; flip angle: 63°).

## MRI data preprocessing

The following preprocessing steps were performed: Motion correction and realignment by registering to the mean EPI, coregistration of the anatomical image and mean EPI using normalized mutual information, segmentation of the T1 image using the unified segmentation approach, and spatial normalization of T1 and EPIs to the Montreal Neurological Institute T1 template (using forward deformations from the segmentation step, voxel size 2*2*2 mm3 for resampling, and 4th Degree B-Spline for interpolation). All functional images were spatially smoothed with an isotropic Gaussian kernel of 6-mm full width at half-maximum.

## Extrastriate body area and fusiform body area mask definition

The bilateral extrastriate body area and right fusiform body area masks were defined as follows: For each of these regions, a search space was defined as a 18 mm sphere around the peak coordinates of the ‘body’ search term in the neurosynth database (www.neurosynth.org), which were masked by a standard grey matter mask (FMRIB Software Library). See figure 1A. For each participant individually, a mask was created within this space by drawing a 8-mm sphere around the peak voxel for the body vs. nature contrast.

## Resting state analysis

Analyses of resting state fMRI data were performed using CONN release 22.v2407 (53) and SPM (54) release 12.7219. Functional and anatomical data were preprocessed using a modular preprocessing pipeline (1) including realignment with correction of susceptibility distortion interactions, slice timing correction, outlier detection, direct segmentation and MNI-space normalization, and smoothing (8 mm full width half maximum). In addition, functional data were denoised using a standard pipeline including the regression of potential confounding effects characterized by white matter timeseries, motion parameters and their first order derivatives outlier scans session effects and their first order derivatives and linear trends within each functional run, followed by bandpass frequency filtering of the BOLD timeseries between 0.008 Hz and 0.09 Hz. CompCor (2) noise components within white matter and CSF were estimated by computing the average BOLD signal as well as the largest principal components orthogonal to the BOLD average, motion parameters, and outlier scans within each subject's eroded segmentation masks.

At the subject level, seed-based connectivity maps and ROI-to-ROI connectivity matrices were estimated characterizing the patterns of functional connectivity with 14 ROIs. These ROIs were: the right EBA, left EBA, and right FBA; 7 salience network ROIs: anterior cingulate cortex, bilateral anterior insula, bilateral supramarginal gyrus, bilateral rostral prefrontal cortex; 4 default mode network ROIs: medial prefrontal cortex, bilateral lateral frontal pole, and posterior cingulate cortex (see supplement for more details).

At the individual level, functional connectivity strength was represented by Fisher-transformed bivariate correlation coefficients from a weighted general linear model, defined separately for each pair of seed and target areas (see main manuscript), modeling the association between their BOLD signal timeseries. Individual scans were weighted by a boxcar signal characterizing each individual task or experimental condition convolved with an SPM canonical hemodynamic response function and rectified.

Group-level analyses were performed using a General Linear Model (GLM). For each individual voxel, a separate GLM was estimated with first-level connectivity measures at this voxel as dependent variables and groups as independent variables. For each seed (right EBA, left EBA, right FBA, defined for each subject individually), 3 separate models were run, testing for (1) a group effect of patients (all treatment groups) vs. controls pre-treatment, (2) an interaction effect of treatment (BPT vs. TAU) and time (pre and post-treatment), (3) an interaction effect of TMS-treatment (TMS vs. sham) vs. treatment as usual) and time (pre and post-treatment).

Voxel-level hypotheses were evaluated using multivariate parametric statistics with random-effects across subjects and sample covariance estimation across multiple measurements. Inferences were performed at the level of individual clusters (groups of contiguous voxels). Cluster-level inferences were based on parametric statistics from Gaussian Random Field theory (3). Results were thresholded using a combination of a cluster-forming *p* < 0.001 voxel-level threshold, and a familywise corrected p-FDR < 0.05 cluster-size threshold. Connection-level hypotheses were evaluated using multivariate parametric statistics with random-effects across subjects and sample covariance estimation across multiple measurements. Inferences were performed at the level of individual clusters (groups of similar connections). Cluster-level inferences were based on parametric statistics within- and between- each pair of networks (Functional Network Connectivity (4)), with networks identified using a complete-linkage hierarchical clustering procedure based on ROI-to-ROI anatomical proximity and functional similarity metrics. Results were thresholded using a combination of a *p* < 0.05 connection-level threshold and a familywise corrected p-FDR < 0.05 cluster-level threshold.

**Body Image Interventions**

The body image interventions were developed by physiotherapists and psychologists with experience of treating eating disorders in psychiatric care. The content of the five sessions was presented in a structured manual with detailed instructions and information on the procedures for each session. Each intervention ended with questions from the therapist, encouraging reflections about body image based on participants’ experiences of the exercises, and potential discrepancies between perceived and actual body size. Each session took between 20-30 minutes to complete.

*Psychoeducation*

The psychoeducation contained information about the body, the anatomy and functions of the skeleton and muscles, and the stomach area, including internal organs, and the size and placing of the bowels and intestines. Participants were presented with a poster of a skeleton with muscles and given oral information. A rope was used to illustrate the intestines.

*Drawing of estimated and actual body size*

Participants drew the contours of their perceived body size on a large piece of paper with positions for head, shoulders, middle of body and arm length marked out. After that, the contour of their actual body size was drawn on the same paper, superimposed on the perceived body size, with the help of the therapist.

*Estimating the size of different body parts using a piece of string*

Participants were encouraged to estimate the size of their waist, overarms and thighs, one at a time, using a piece of string. The therapist then measured the actual size using a differently colored piece of string and the two pieces of string (perceived and actual size) were superimposed on each other forming two circles.

*Estimating the size of different body parts using different sized hula hoop rings*

Using eight hula hoop rings of different sizes, participants were instructed to choose the one they perceived would fit exactly round their body. Participants then tried fitting their body in the ring they had chosen. Based on this feedback experience and the discussion that followed, participants were encouraged to choose a new ring and try fitting that size over their body.

*Adjusting the size of a computerized morph of their body*

Participants were instructed to adjust a computerized body morph to the size they perceived their own body to be. The body morph was presented on a computer screen. The different versions were shown to the participants at the end of week 4.

# Supplementary Results

## Effect of body perception training and TMS on clinical outcome

*Body shape Questionnaire (primary outcome)*

The results are described in the main manuscript, but added here for completeness. Both the body perception training (BPT) and treatment-as-usual (TAU) group showed a decrease in BSQ-scores from pre- to post-treatment, *F*(1,38) = 14.22, *p* < .001. Descriptively, this was stronger for the BPT than for the TAU group, but this interaction effect (treatment x time) was not significant *F*(1,38) = 3.92, *p* = .055).

For both TMS-treatment groups (within the BPT group), BSQ-scores decreased from pre- to post-treatment, *F*(1,18) = 15.259, *p* = .001. No TMS effect was found for this first time-interval (pre- to post-treatment), *F*(1,18) = 2.17, *p* = .16. Comparing all 3 treatment groups (TAU, BPT-TMS active, BPT-TMS sham) across all 3 timepoints, there was a significant effect of treatment type (treatment x time interaction): *F*(4,70) = 3.62, *p* = .010 (see main manuscript). Post-hoc tests (Holm-correction) indicated a significant decrease in BSQ scores in the active TMS group from pre- to post-treament (*p* = .018), and from pre-treament to 6-months follow-up (*p* = .019). In the TAU group, there was a significant decrease from pre-treatment to 6-months follow-up (*p* = .037).

**Table S1** Post-hoc comparisons for BSQ. P-value and confidence intervals are adjusted for comparing a family of 36 estimates.

|  | | | 95% CI for Mean Difference | |  | | | 95% CI for Cohen's d | |  |
| --- | --- | --- | --- | --- | --- | --- | --- | --- | --- | --- |
|  |  | Mean Difference | Lower | Upper | SE | t | Cohen's d | Lower | Upper | p_holm_ |
| TMS BPT, pre | sham BPT, pre | 16.767 | -31.379 | 64.913 | 13.863 | 1.209 | 0.514 | -0.977 | 2.004 | 1 |
|  | TAU, pre | 10.719 | -31.683 | 53.121 | 12.209 | 0.878 | 0.328 | -0.978 | 1.634 | 1 |
|  | TMS BPT, post | 32.444 | 3.001 | 61.888 | 8.478 | 3.827 | 0.994 | 0.002 | 1.986 | 0.018 |
|  | sham BPT, post | 31.067 | -19.413 | 81.546 | 14.535 | 2.137 | 0.952 | -0.644 | 2.548 | 1 |
|  | TAU, post | 17.982 | -25.824 | 61.789 | 12.614 | 1.426 | 0.551 | -0.81 | 1.912 | 1 |
|  | TMS BPT, 6-mo | 37 | 3.206 | 70.794 | 9.73 | 3.802 | 1.133 | -0.004 | 2.271 | 0.019 |
|  | sham BPT, 6-mo | 18.367 | -33.108 | 69.841 | 14.821 | 1.239 | 0.563 | -1.031 | 2.157 | 1 |
|  | TAU, 6-mo | 34.561 | -9.851 | 78.974 | 12.788 | 2.703 | 1.059 | -0.371 | 2.489 | 0.337 |
|  |  |  |  |  |  |  |  |  |  |  |
| sham BPT, pre | TAU, pre | -6.047 | -46.985 | 34.891 | 11.788 | -0.513 | -0.185 | -1.442 | 1.071 | 1 |
|  | TMS BPT, post | 15.678 | -35.054 | 66.41 | 14.608 | 1.073 | 0.48 | -1.087 | 2.047 | 1 |
|  | sham BPT, post | 14.3 | -13.632 | 42.232 | 8.043 | 1.778 | 0.438 | -0.437 | 1.313 | 1 |
|  | TAU, post | 1.216 | -41.176 | 43.607 | 12.206 | 0.1 | 0.037 | -1.261 | 1.336 | 1 |
|  | TMS BPT, 6-mo | 20.233 | -31.598 | 72.064 | 14.924 | 1.356 | 0.62 | -0.989 | 2.228 | 1 |
|  | sham BPT, 6-mo | 1.6 | -30.46 | 33.66 | 9.231 | 0.173 | 0.049 | -0.933 | 1.031 | 1 |
|  | TAU, 6-mo | 17.795 | -25.222 | 60.811 | 12.386 | 1.437 | 0.545 | -0.792 | 1.882 | 1 |
|  |  |  |  |  |  |  |  |  |  |  |
| TAU, pre | TMS BPT, post | 21.725 | -23.592 | 67.042 | 13.048 | 1.665 | 0.666 | -0.75 | 2.081 | 1 |
|  | sham BPT, post | 20.347 | -23.311 | 64.006 | 12.571 | 1.619 | 0.623 | -0.739 | 1.986 | 1 |
|  | TAU, post | 7.263 | -13.001 | 27.527 | 5.835 | 1.245 | 0.223 | -0.405 | 0.85 | 1 |
|  | TMS BPT, 6-mo | 26.281 | -20.263 | 72.825 | 13.402 | 1.961 | 0.805 | -0.659 | 2.27 | 1 |
|  | sham BPT, 6-mo | 7.647 | -37.158 | 52.453 | 12.901 | 0.593 | 0.234 | -1.142 | 1.61 | 1 |
|  | TAU, 6-mo | 23.842 | 0.584 | 47.101 | 6.697 | 3.56 | 0.73 | -0.044 | 1.505 | 0.037 |
|  |  |  |  |  |  |  |  |  |  |  |
| TMS BPT, post | sham BPT, post | -1.378 | -54.33 | 51.574 | 15.247 | -0.09 | -0.042 | -1.664 | 1.58 | 1 |
|  | TAU, post | -14.462 | -61.096 | 32.172 | 13.428 | -1.077 | -0.443 | -1.883 | 0.997 | 1 |
|  | TMS BPT, 6-mo | 4.556 | -25.491 | 34.602 | 8.651 | 0.527 | 0.14 | -0.783 | 1.062 | 1 |
|  | sham BPT, 6-mo | -14.078 | -67.979 | 39.823 | 15.52 | -0.907 | -0.431 | -2.092 | 1.23 | 1 |
|  | TAU, 6-mo | 2.117 | -45.086 | 49.32 | 13.592 | 0.156 | 0.065 | -1.381 | 1.511 | 1 |
|  |  |  |  |  |  |  |  |  |  |  |
| sham BPT, post | TAU, post | -13.084 | -58.108 | 31.94 | 12.964 | -1.009 | -0.401 | -1.79 | 0.988 | 1 |
|  | TMS BPT, 6-mo | 5.933 | -48.072 | 59.939 | 15.55 | 0.382 | 0.182 | -1.474 | 1.838 | 1 |
|  | sham BPT, 6-mo | -12.7 | -41.204 | 15.804 | 8.207 | -1.547 | -0.389 | -1.277 | 0.499 | 1 |
|  | TAU, 6-mo | 3.495 | -42.119 | 49.108 | 13.134 | 0.266 | 0.107 | -1.291 | 1.505 | 1 |
|  |  |  |  |  |  |  |  |  |  |  |
| TAU, post | TMS BPT, 6-mo | 19.018 | -28.81 | 66.845 | 13.771 | 1.381 | 0.583 | -0.902 | 2.068 | 1 |
|  | sham BPT, 6-mo | 0.384 | -45.753 | 46.521 | 13.285 | 0.029 | 0.012 | -1.402 | 1.425 | 1 |
|  | TAU, 6-mo | 16.579 | -4.1 | 37.258 | 5.954 | 2.784 | 0.508 | -0.16 | 1.176 | 0.284 |
|  |  |  |  |  |  |  |  |  |  |  |
| TMS BPT, 6-mo | sham BPT, 6-mo | -18.633 | -73.57 | 36.304 | 15.818 | -1.178 | -0.571 | -2.27 | 1.129 | 1 |
|  | TAU, 6-mo | -2.439 | -50.821 | 45.944 | 13.931 | -0.175 | -0.075 | -1.557 | 1.408 | 1 |
|  |  |  |  |  |  |  |  |  |  |  |
| sham BPT, 6-mo | TAU, 6-mo | 16.195 | -30.517 | 62.907 | 13.45 | 1.204 | 0.496 | -0.95 | 1.942 | 1 |

### *Body Attitude Test*

The BPT group showed a stronger decrease than the TAU on the BAT from pre- to post-treatment (time x group interaction: F(1,38) = 12.43, *p* = .001). For both TMS-treatment groups (within the BPT group), BAT-scores decreased from pre- to post-treatment, F(1,18) = 21.21, *p* < .001. No TMS effect was found for this first time-interval (pre- to post-treatment), F(1,18) = 2.53, *p* = .13. Comparing all 3 treatment groups (TAU, BPT-TMS active, BPT-TMS sham) across all 3 timepoints, there was a significant effect of treatment type (treatment x time interaction): F(4,70) = 4.12, *p* = .005. Post-hoc tests (Holm-correction) indicated a significant decrease in BAT scores in the active TMS group from pre- to post-treatment (p = .001).

**Table S2**. Post-hoc comparisons for BAT P-value and confidence intervals are adjusted for comparing a family of 36 estimates.

|  | | | 95% CI for Mean Difference | |  | | | 95% CI for Cohen's d | |  |
| --- | --- | --- | --- | --- | --- | --- | --- | --- | --- | --- |
|  |  | Mean Difference | Lower | Upper | SE | t | Cohen's d | Lower | Upper | p_holm_ |
| TMS BPT, pre | sham BPT, pre | 5.5 | -19.072 | 30.07 | 7.075 | 0.777 | 0.315 | -1.099 | 1.729 | 1 |
|  | TAU, pre | 6.053 | -15.588 | 27.69 | 6.231 | 0.971 | 0.347 | -0.901 | 1.595 | 1 |
|  | TMS BPT, post | 18.778 | 5.035 | 32.52 | 3.957 | 4.745 | 1.076 | 0.171 | 1.981 | 0.001 |
|  | sham BPT, post | 14.4 | -11.818 | 40.62 | 7.549 | 1.908 | 0.825 | -0.716 | 2.365 | 1 |
|  | TAU, post | 6.158 | -16.476 | 28.79 | 6.517 | 0.945 | 0.353 | -0.952 | 1.658 | 1 |
|  | TMS BPT, 6-mo | 17.222 | -0.644 | 35.09 | 5.144 | 3.348 | 0.987 | -0.116 | 2.089 | 0.067 |
|  | sham BPT, 6-mo | 10.4 | -17.256 | 38.06 | 7.963 | 1.306 | 0.596 | -1.008 | 2.199 | 1 |
|  | TAU, 6-mo | 17.316 | -6.202 | 40.83 | 6.772 | 2.557 | 0.992 | -0.417 | 2.401 | 0.481 |
|  |  |  |  |  |  |  |  |  |  |  |
| sham BPT, pre | TAU, pre | 0.553 | -20.341 | 21.45 | 6.016 | 0.092 | 0.032 | -1.165 | 1.229 | 1 |
|  | TMS BPT, post | 13.278 | -13.117 | 39.67 | 7.6 | 1.747 | 0.761 | -0.784 | 2.305 | 1 |
|  | sham BPT, post | 8.9 | -4.137 | 21.94 | 3.754 | 2.371 | 0.51 | -0.266 | 1.286 | 0.725 |
|  | TAU, post | 0.658 | -21.263 | 22.58 | 6.312 | 0.104 | 0.038 | -1.218 | 1.294 | 1 |
|  | TMS BPT, 6-mo | 11.722 | -16.255 | 39.7 | 8.056 | 1.455 | 0.672 | -0.955 | 2.298 | 1 |
|  | sham BPT, 6-mo | 4.9 | -12.049 | 21.85 | 4.88 | 1.004 | 0.281 | -0.697 | 1.259 | 1 |
|  | TAU, 6-mo | 11.816 | -11.016 | 34.65 | 6.574 | 1.797 | 0.677 | -0.661 | 2.015 | 1 |
|  |  |  |  |  |  |  |  |  |  |  |
| TAU, pre | TMS BPT, post | 12.725 | -10.964 | 36.41 | 6.821 | 1.866 | 0.729 | -0.661 | 2.119 | 1 |
|  | sham BPT, post | 8.347 | -14.459 | 31.15 | 6.567 | 1.271 | 0.478 | -0.843 | 1.8 | 1 |
|  | TAU, post | 0.105 | -9.353 | 9.564 | 2.723 | 0.039 | 0.006 | -0.536 | 0.548 | 1 |
|  | TMS BPT, 6-mo | 11.17 | -14.271 | 36.61 | 7.325 | 1.525 | 0.64 | -0.842 | 2.121 | 1 |
|  | sham BPT, 6-mo | 4.347 | -20.098 | 28.79 | 7.039 | 0.618 | 0.249 | -1.155 | 1.653 | 1 |
|  | TAU, 6-mo | 11.263 | -1.033 | 23.56 | 3.541 | 3.181 | 0.645 | -0.108 | 1.399 | 0.101 |
|  |  |  |  |  |  |  |  |  |  |  |
| TMS BPT, post | sham BPT, post | -4.378 | -32.31 | 23.56 | 8.043 | -0.544 | -0.251 | -1.854 | 1.353 | 1 |
|  | TAU, post | -12.62 | -37.22 | 11.98 | 7.083 | -1.782 | -0.723 | -2.164 | 0.718 | 1 |
|  | TMS BPT, 6-mo | -1.556 | -18.091 | 14.98 | 4.761 | -0.327 | -0.089 | -1.037 | 0.859 | 1 |
|  | sham BPT, 6-mo | -8.378 | -37.664 | 20.91 | 8.433 | -0.993 | -0.48 | -2.169 | 1.21 | 1 |
|  | TAU, 6-mo | -1.462 | -26.877 | 23.95 | 7.318 | -0.2 | -0.084 | -1.54 | 1.373 | 1 |
|  |  |  |  |  |  |  |  |  |  |  |
| sham BPT, post | TAU, post | -8.242 | -31.993 | 15.51 | 6.839 | -1.205 | -0.472 | -1.847 | 0.902 | 1 |
|  | TMS BPT, 6-mo | 2.822 | -26.611 | 32.26 | 8.475 | 0.333 | 0.162 | -1.526 | 1.849 | 1 |
|  | sham BPT, 6-mo | -4 | -19.687 | 11.69 | 4.517 | -0.886 | -0.229 | -1.133 | 0.675 | 1 |
|  | TAU, 6-mo | 2.916 | -21.678 | 27.51 | 7.082 | 0.412 | 0.167 | -1.244 | 1.578 | 1 |
|  |  |  |  |  |  |  |  |  |  |  |
| TAU, post | TMS BPT, 6-mo | 11.064 | -15.227 | 37.36 | 7.57 | 1.462 | 0.634 | -0.895 | 2.163 | 1 |
|  | sham BPT, 6-mo | 4.242 | -21.087 | 29.57 | 7.293 | 0.582 | 0.243 | -1.211 | 1.698 | 1 |
|  | TAU, 6-mo | 11.158 | -0.223 | 22.54 | 3.277 | 3.405 | 0.639 | -0.065 | 1.343 | 0.059 |
|  |  |  |  |  |  |  |  |  |  |  |
| TMS BPT, 6-mo | sham BPT, 6-mo | -6.822 | -37.543 | 23.9 | 8.846 | -0.771 | -0.391 | -2.158 | 1.376 | 1 |
|  | TAU, 6-mo | 0.094 | -26.962 | 27.15 | 7.79 | 0.012 | 0.005 | -1.545 | 1.555 | 1 |
|  |  |  |  |  |  |  |  |  |  |  |
| sham BPT, 6-mo | TAU, 6-mo | 6.916 | -19.206 | 33.04 | 7.521 | 0.919 | 0.396 | -1.109 | 1.902 | 1 |

*EDE-Q*

Both the BPT group and TAU group showed a decrease in EDE-Q-scores from pre- to post-treatment, *F*(1,38) = 7.09, *p* = .011. There was no significant evidence for a stronger decrease in either treatment group (treatment x time: *F*(1,38) = .051, *p* = .82). For both TMS-treatment groups (within the BPT group) specifically, EDE-Q scores did not decrease significantly during this first time-interval (pre-to post-treatment), *F*(1,18) = 3.18, *p* = .091, and no TMS-effect was found, *F*(1,18) = 0.12, *p* = .73. Comparing all 3 treatment groups (TAU, BPT-TMS active, BPT-TMS sham) across all 3 timepoints, there was a significant overall decrease in EDE-Q across all groups, *F*(2,70) = 9.653, *p* < .001, but no effect of treatment on this decrease (treatment x time interaction: Time*Group *F*(4,70) = 0.22, *p* = .93).

*Figure rating scale*

There were 3 figure rating scales (FRS): Think, Feel and Ideal.

For FRS-think, the BPT group showed a stronger decrease than the TAU on the BAT from pre- to post-treatment (time x group interaction: F(1,37) = 6.01, *p* = .019). For both TMS-treatment groups (within the BPT group), FRS-think scores decreased from pre- to post-treatment, F(1,17) = 6.78, *p* = .019. No TMS effect was found for this first time-interval (pre- to post-treatment), F(1,17) = 6.78, *p* = .019. Comparing all 3 treatment groups (TAU, BPT-TMS active, BPT-TMS sham) across all 6 timepoints (pre-treatment, week 1,week 2, week 3, week 4/post-treatment, follow-up), there was a significant overall decrease in FRS-think across all groups, F(5,150) = 2.77, *p* = .020, but no effect of treatment on this decrease (F(10,150) = 1.71, *p* = .082).

For FRS-feel, both the BPT group and TAU group showed a decrease in scores from pre- to post-treatment, F(1,38) = 12.47, *p* = .001. There was no significant evidence for a stronger decrease in either treatment group (treatment x time: F(1,38) = 1.18, *p* = 0.28). For both TMS-treatment groups (within the BPT group), FRS-feel scores decreased from pre- to post-treatment, F(1,18) = 11.56, *p* = .003. No TMS effect was found for this first time-interval (pre- to post-treatment), F(1,18) = 1.28, *p* = 0.27. Comparing all 3 treatment groups (TAU, BPT-TMS active, BPT-TMS sham) across all 6 timepoints (pre-treatment, week 1,week 2, week 3, week 4/post-treatment, follow-up), there was a significant overall decrease in FRS-feel across all groups, F(5,155) = 3.81, *p* = .003, but no effect of treatment on this decrease (F(10,155) = 0.58, *p* = .83).

For FRS-ideal, both the BPT group and TAU group showed an increase in scores from pre- to post-treatment, F(1,36) = 9.97, *p* = .003. There was no significant evidence for a stronger decrease in either treatment group (treatment x time: F(1,38) = 0.35, *p* = .56). For both TMS-treatment groups (within the BPT group), FRS-ideal scores increased from pre- to post-treatment, F(1,18) = 1.17, *p* = 0.30. No TMS effect was found for this first time-interval (pre- to post-treatment), F(1,18) = 1.28, *p* = 0.27. Comparing all 3 treatment groups (TAU, BPT-TMS active, BPT-TMS sham) across all 6 timepoints (pre-treatment, week 1,week 2, week 3, week 4/post-treatment, follow-up), there was a significant overall decrease in FRS-ideal across all groups, F(5,150) = 6.63, *p* < .001, but no effect of treatment on this decrease (F(10,150) = 0.49, *p* = .90).

*BMI*

Neither the BPT group nor the TAU group showed an increase in BMI from pre- to post-treatment, F(1,35) = .40, *p* = .53. There was no significant evidence for a stronger increase in either treatment group (treatment x time: F(1,35) = .06, *p* = .80). For both TMS-treatment groups (within the BPT group) specifically, BMI did not increase significantly during this first time-interval (pre-to post-treatment), F(1,18) = 0.11, *p* = .75, and no TMS-effect was found, F(1,18) = 0.16, *p* = .70. Comparing all 3 treatment groups (TAU, BPT-TMS active, BPT-TMS sham) across all 3 timepoints, there was no significant overall increase in BMI across all groups, F(2,66) = 2.87, *p* = .064. However, this increase was significant when one outlier at the follow-up measurement was removed (p = .009). There was no effect of treatment on this increase (treatment x time interaction: Time*Group F(4,66) = 0.21, *p* = .93).

## EBA localizer task

**Masks (search space)**

**
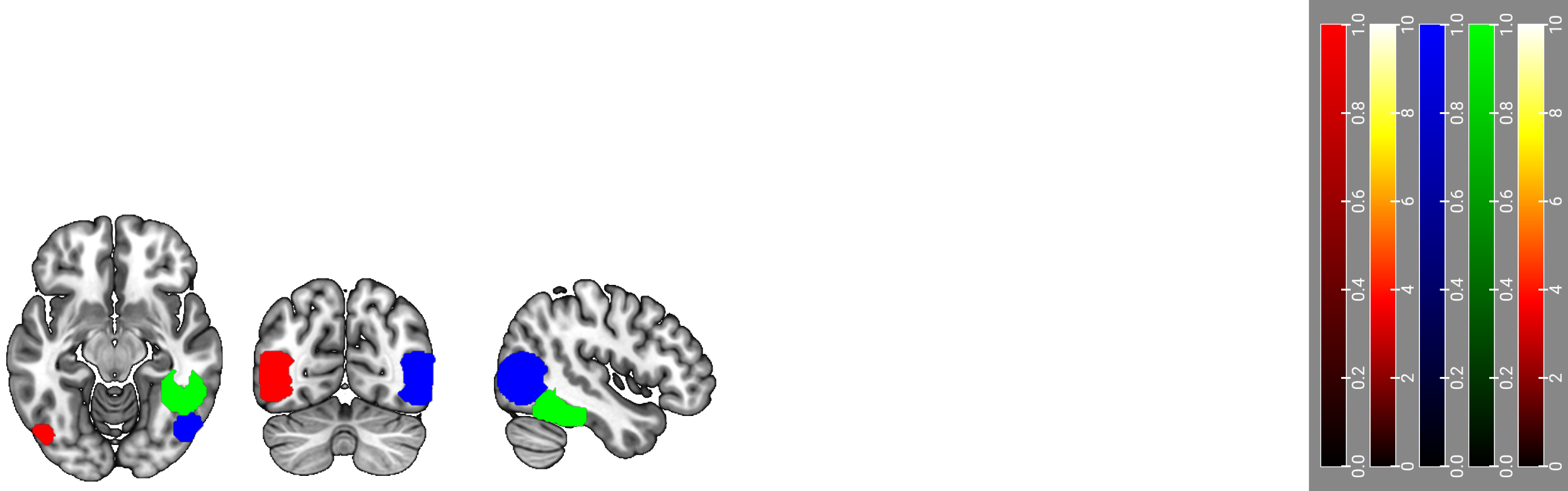
**

**Z = -12**

**Y = -69**

**X = 45**

**Patients: Body vs. Nature**


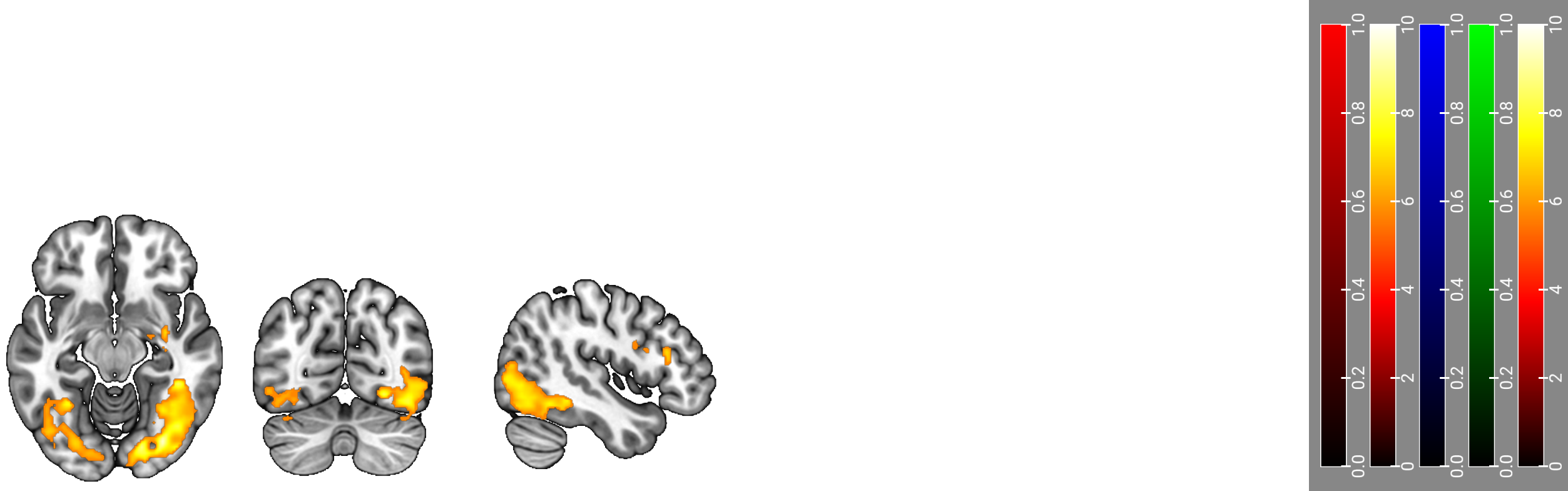


**Controls: Body vs. Nature**

**
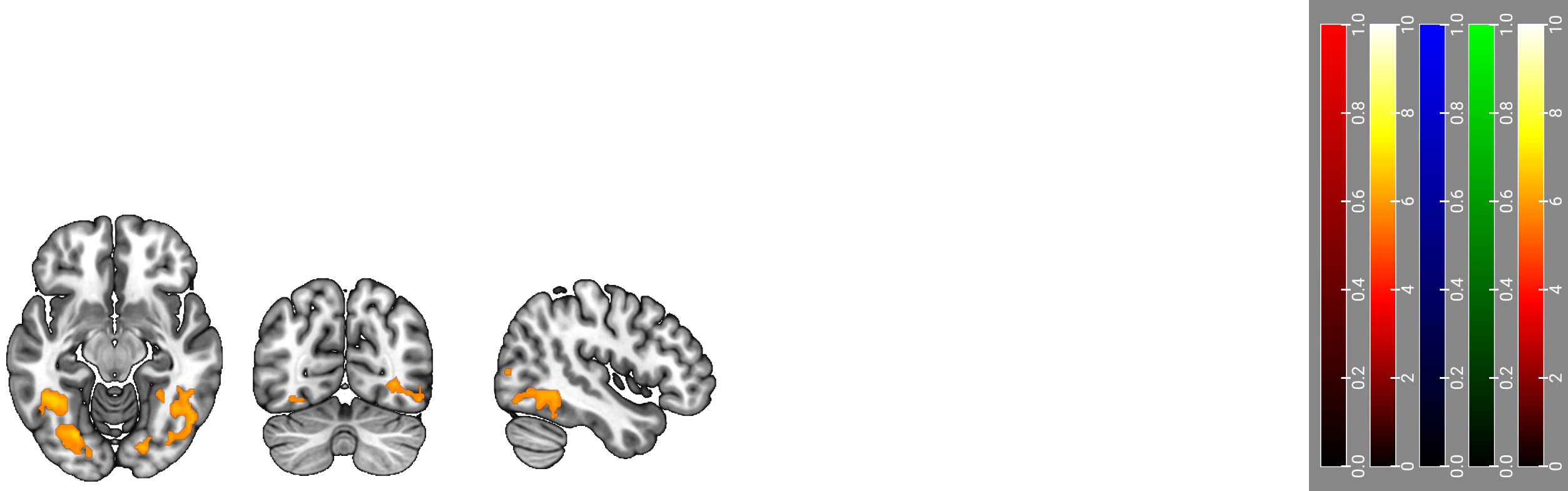
**

**T**

**
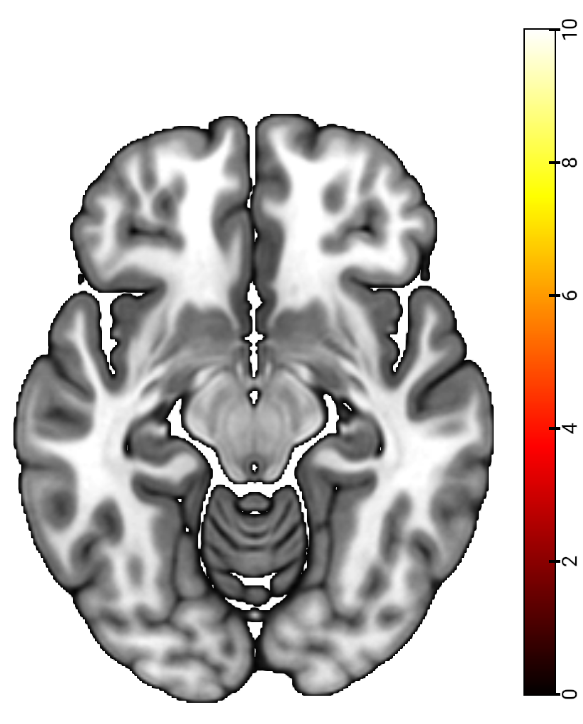
**

**Figure S1. Activation for Body vs. Nature pictures. A** Regions of interest: left extrastriate body area (red), right extrastriate body area (blue), right fusiform body area (green). **B.** Anorexia patients showed increased activation in the rFBA and bilateral EBA for body images compared to nature images. **C.** Healthy controls showed a similar pattern. All images in neurological orientation and thresholded at *p* < .05 (family-wise error corrected). T = t-value. X,Y,Z according to Montreal neurological institute coordinate system.

An exploratory ROI analysis on mean activation around individual peak activation within the bilateral EBA (i.e. mean beta estimates extracted from 8-mm spheres around the individual peak voxel within a predefined EBA mask) revealed no difference in activation between patients and controls at baseline. However, mean activation around individual peak activation within the right FBA was higher for the patients (*p* = .041)

## Self-other-touch task

Pre-treatment, AN patients showed increased activation for other vs. self(corrected for movement) in the bilateral parietal operculum (S2), bilateral temporal poles, and insula, comparable to previous work with healthy controls (5, 6) (Figure 4A in main manuscript). Directly compared to the healthy control group, no differences were found at whole-brain corrected level. However, small-volume corrected (SVC) analyses showed decreased activation for anorexia patients, when compared to healthy controls, for other- vs. self(corrected for movement)-touch in the right anterior cingulate cortex (ACC), right superior temporal gyrus (STG) as well as the right fusiform gyrus (FG)/EBA and right FBA (table S3). No differences were found for the left EBA and right insula (note that this analysis was restricted to right-sided volumes of interest, except for the left EBA). Note that the rFG/rEBA cluster overlaps with the EBA mask, but that its peak is in a different location than the rEBA cluster showing treatment effects over time (MNI_xyz_ = 38, -80, 10; see main manuscript and figure S3)

Table S3. Significantly increased activation for other vs. self-touch when comparing healthy controls with anorexia patients (Figure 4BCD in main manuscript)

|  | **laterality** | **MNI(x,y,z)** | | | ***p*-FWE (peak)** |
| --- | --- | --- | --- | --- | --- |
| Anterior cingulate cortex | R | 8 | 44 | 4 | .010 |
| Superior temporal gyrus | R | 68 | -14 | 8 | .029 |
| Fusiform gyrus/EBA | R | 36 | -70 | -8 | .026 |
| FBA | R | 40 | -40 | -20 | .003 |

**TMS-sham TMS-active Treatment as usual**


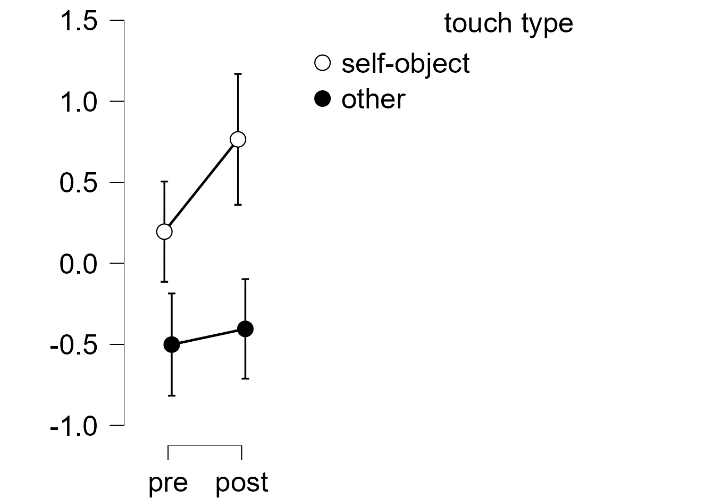

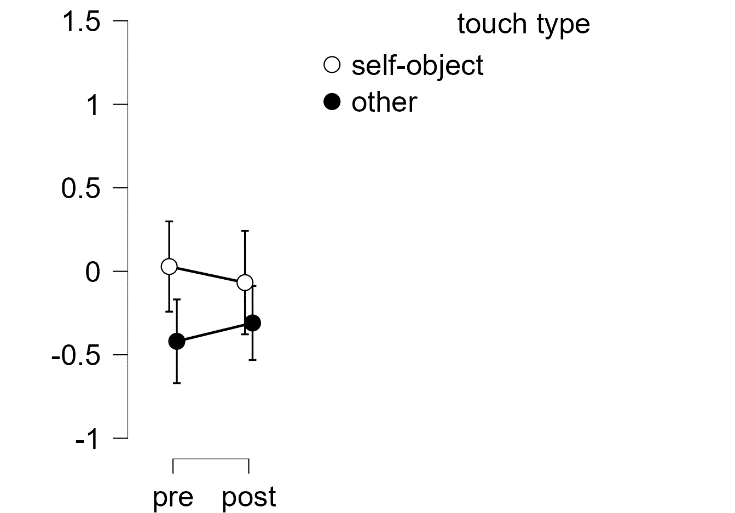

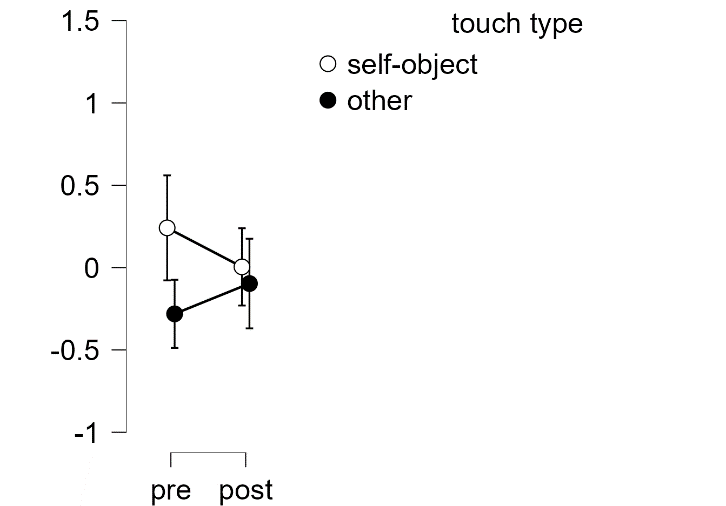


**Pre-treatment**


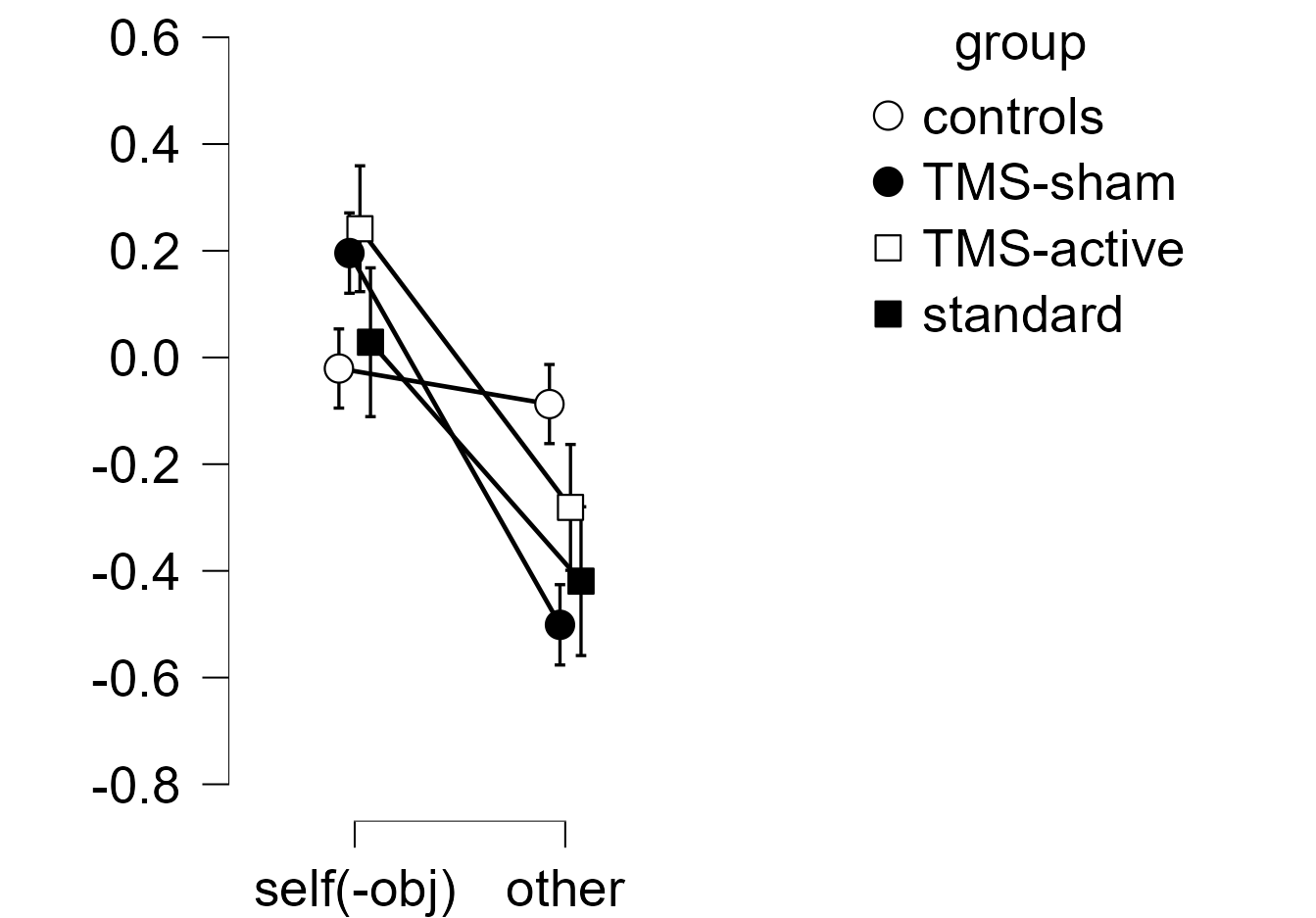


**Figure S2** Right EBA cluster activation before and after treatment. The difference between self- and-other-touch decreased for the active TMS group (similar pattern for TAU group), but increased for the sham-TMS group. Pre-treatment, all patients groups show increased activation for self vs. other touch in this specific region, whereas controls did not show this effect. Beta-values are extracted from the significant TMS-treatment effect cluster (contrast image: POST: [ [active TMS: other-touch > self-touch] > [passive TMS: other-touch > self-touch]] > PRE: [ [active TMS: other-touch > self-touch] > [passive TMS: other-touch > self-touch]]; see main manuscript)


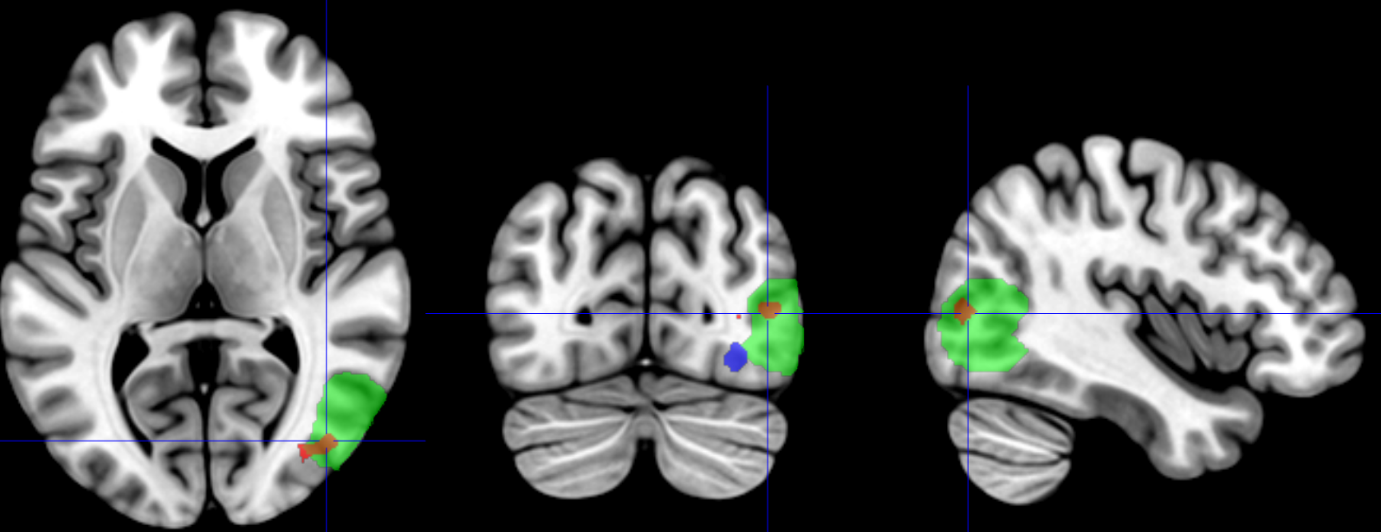


Right EBA mask

rEBA treatment effect

rFG/rEBA group effect at baseline

**Figure S3**. Specific location of rFG/rEBA group effect (MNI_xyz_ = 36, -70, -8) and rEBA treatment effect (MNI_xyz_ = 38, -80, 10) . rFG = right fusiform gyrus, rEBA = right EBA.

## Resting state

*Seed-to-voxel analysis*

Compared to controls, AN patients showed stronger connectivity between the left EBA (defined for each participant individually based on the EBA localizer task) and the right superior parietal lobule pre-treatment (Figure S4A; Table S4). No pre-treatment differences between patients and controls were found for right EBA and right FBA connectivity. After treatment, connectivity between the right EBA and clusters in the bilateral frontal pole and left cerebellum decreased compared to before treatment. Similar decreases in connectivity were found between the left EBA and a similar cluster in the left cerebellum as well as the bilateral precentral gyrus/inferior frontal gyrus (Figure S4A; Table S4).

**Table S4**. Significant clusters of activation from resting state seed-to-voxel analyses

|  |  | **laterality** | **MNI(x,y,z)** | | | ***k*** | ***p*-FWE (size)** |
| --- | --- | --- | --- | --- | --- | --- | --- |
| *Anorexia patients > healthy controls: left EBA connectivity* | | | | | |  |  |
|  | Superior parietal lobule | R | 38 | -52 | 54 | 380 | 0.003 |
| *Anorexia patients: left EBA connectivity decrease after treatment* | | | | | | |  |
|  | Cerebellum | L | -24 | -72 | -44 | 343 | 0.001 |
|  | Inferior Frontal Gyrus / Precentral gyrus | L | -48 | 8 | 22 | 256 | 0.006 |
|  | Inferior Frontal Gyrus | R | 50 | 10 | 24 | 171 | 0.039 |
|  |  |  |  |  |  |  |  |
| *Anorexia patients: right EBA connectivity decrease after treatment* | | | | | | |  |
|  | Cerebellum | L | -26 | -70 | -44 | 590 | 0.000 |
|  | Frontal Pole | L | -38 | 62 | 8 | 247 | 0.008 |
|  | Frontal Pole | R | 38 | 30 | 22 | 236 | 0.010 |

*ROI-to-ROI analysis*

When comparing AN patients before treatment to healthy controls, several of the pre-selected regions including the right FBA, left EBA, and right EBA showed stronger connectivity to a cluster of salience network regions, including the anterior insula, ACC, SMG and rostral prefrontal cortex (Figure 5B ). There was no effect of time (pre- and post-treatment) or interaction between time and treatment group.

**A**

**Anorexia patients > healthy controls**

Left EBA seed


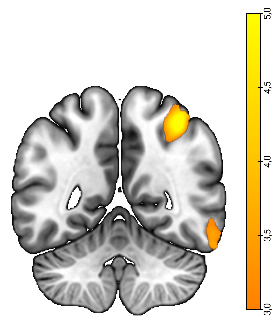


**Anorexia patients:**

**pre- vs. post-treatment**

Left EBA seed

**Anorexia patients:**

**pre- vs. post-treatment**

Right EBA seed


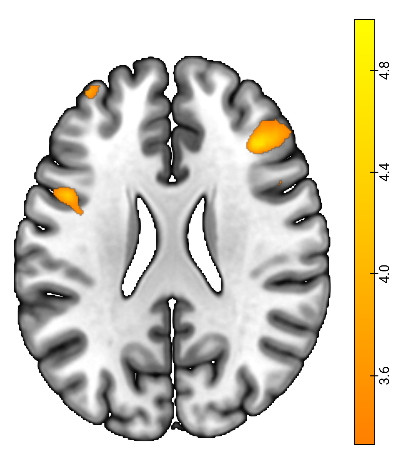

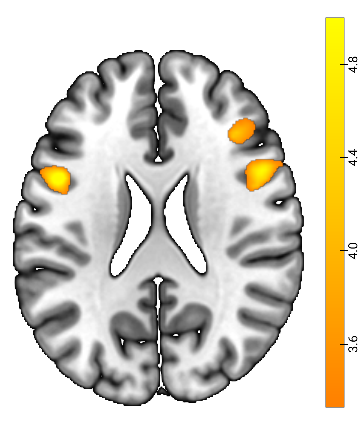


**B**


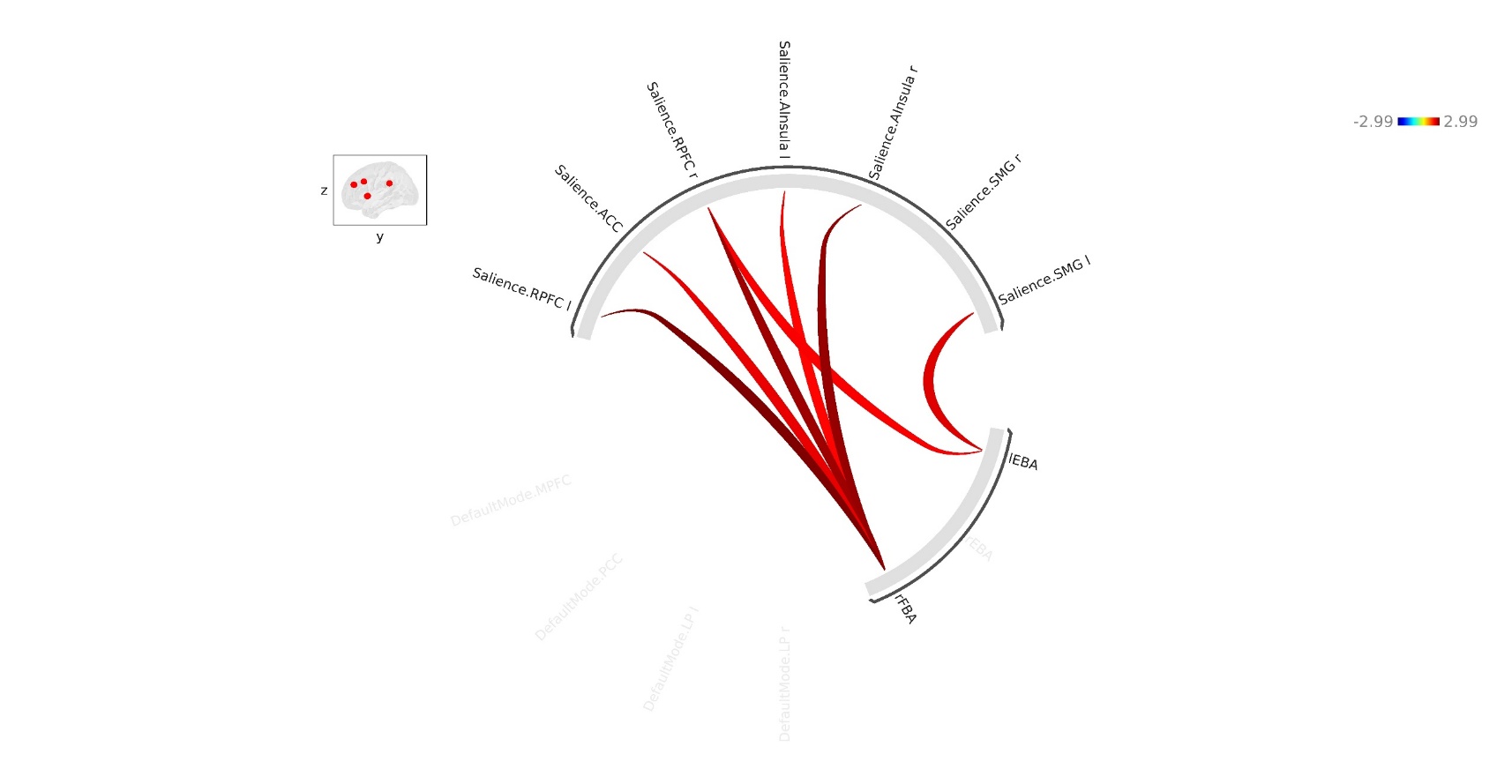


**Figure S4 Resting state connectivity at baseline and after treatment A.** Seed-based analyses results. Top: Increased connectivity between the left EBA and superior parietal lobule for anorexia patients compared to controls. Bottom: Decreased connectivity between the left and right EBA and frontal clusters (see table S3) after treatment in anorexia patients (across all treatment groups) All images thresholded at *p* < .001 uncorrected for visualization.  **B**. ROI-to-ROI analysis results. A cluster of ROIs containing the right FBA, right EBA and left EBA showed increased connectivity with the salience network in anorexia patients (pre-treatment) compared to healthy controls. RPFC = rostral prefrontal cortex, SMG = supra-marginal gyrus. Results are significant at the cluster level (i.e. connectivity between groups of ROIs). Red lines indicate the strongest ROI-ROI connections.

**References**

1. Nieto-Castanon A, Whitfield-Gabrieli S (2021): CONN functional connectivity toolbox (RRID: SCR_009550), Version 21. *Series CONN functional connectivity toolbox (RRID: SCR_009550), Version*. 21.

2. Behzadi Y, Restom K, Liau J, Liu TT (2007): A component based noise correction method (CompCor) for BOLD and perfusion based fMRI. *Neuroimage*. 37:90-101.

3. Worsley KJ, Marrett S, Neelin P, Vandal AC, Friston KJ, Evans AC (1996): A unified statistical approach for determining significant signals in images of cerebral activation. *Human brain mapping*. 4:58-73.

4. Jafri MJ, Pearlson GD, Stevens M, Calhoun VD (2008): A method for functional network connectivity among spatially independent resting-state components in schizophrenia. *Neuroimage*. 39:1666-1681.

5. Boehme R, Hauser S, Gerling GJ, Heilig M, Olausson H (2019): Distinction of self-produced touch and social touch at cortical and spinal cord levels. *PNAS Proceedings of the National Academy of Sciences*

6. Kaldewaij R, Salamone PC, Enmalm A, Östman L, Pietrzak M, Karlsson H, et al. (2024): Ketamine reduces the neural distinction between self-and other-produced affective touch: a randomized double-blind placebo-controlled study. *Neuropsychopharmacology*. 49:1767-1774.
